# Efficiency in Rule- vs. Plan-Based Movements Is Modulated by Action-Mode

**DOI:** 10.3389/fpsyg.2018.00309

**Published:** 2018-03-13

**Authors:** Jean P. P. Scheib, Sarah Stoll, J. Lukas Thürmer, Jennifer Randerath

**Affiliations:** ^1^Department of Psychology, University of Konstanz, Konstanz, Germany; ^2^Department of Political Science and Administration, University of Konstanz, Konstanz, Germany; ^3^Department of Psychology, University of Pittsburgh, Pittsburgh, PA, United States; ^4^Lurija Institute for Rehabilitation Science and Health Research, Kliniken Schmieder, Allensbach, Germany

**Keywords:** grasping, action planning, implementation intentions, end-state comfort, pantomime, drift diffusion, motor cognition

## Abstract

The rule/plan motor cognition (RPMC) paradigm elicits visually indistinguishable motor outputs, resulting from either plan- or rule-based action-selection, using a combination of essentially interchangeable stimuli. Previous implementations of the RPMC paradigm have used pantomimed movements to compare plan- vs. rule-based action-selection. In the present work we attempt to determine the generalizability of previous RPMC findings to real object interaction by use of a grasp-to-rotate task. In the plan task, participants had to use prospective planning to achieve a comfortable post-handle rotation hand posture. The rule task used implementation intentions (if-then rules) leading to the same comfortable end-state. In Experiment A, we compare RPMC performance of 16 healthy participants in pantomime and real object conditions of the experiment, within-subjects. Higher processing efficiency of rule- vs. plan-based action-selection was supported by diffusion model analysis. Results show a significant response-time increase in the pantomime condition compared to the real object condition and a greater response-time advantage of rule-based vs. plan-based actions in the pantomime compared to the real object condition. In Experiment B, 24 healthy participants performed the real object RPMC task in a task switching vs. a blocked condition. Results indicate that plan-based action-selection leads to longer response-times and less efficient information processing than rule-based action-selection in line with previous RPMC findings derived from the pantomime action-mode. Particularly in the task switching mode, responses were faster in the rule compared to the plan task suggesting a modulating influence of cognitive load. Overall, results suggest an advantage of rule-based action-selection over plan-based action-selection; whereby differential mechanisms appear to be involved depending on the action-mode. We propose that cognitive load is a factor that modulates the advantageous effect of implementation intentions in motor cognition on different levels as illustrated by the varying speed advantages and the variation in diffusion parameters per action-mode or condition, respectively.

## 1. Introduction

The development of human interaction with physical tools and objects has come a long way from grasping and using basic stone tools to modern-day video game controllers. Aside from the usage of tools and objects, the grasping movement is a manual skill component for which cognition plays a significant role, as will be detailed below. Considering the multitude of possibilities for grasping an object (variable macroscopic features of grasping include grip force, hand posture, hand shape, and placement on the object), why do we select the grasps that we do?

Healthy individuals pick up a given object with a grip that matches the properties of the object, such as size, weight, and surface texture (Cadoret and Smith, 1996; Flanagan and Wing, 1997; Hermsdörfer et al., 2008; Li et al., 2009). For example, when picking up an iron bar, knowledge of iron's weight characteristics leads to a tight initial grip, as iron is known to be relatively heavy. Grip force can be further mediated by the bar's surface texture, which might be smooth or rough-textured. Smooth textures typically lead to a tighter grip to hinder the object from slipping.

In addition to physical properties, the way we grasp can be influenced by the subsequent action intended for the object (Rosenbaum et al., 1990; Stelmach et al., 1994; Zhang and Rosenbaum, 2008). The phenomenon of adopting an initially uncomfortable hand posture to achieve a biomechanically comfortable end-state is known as the *end-state comfort effect* (see Rosenbaum et al., 2012 for an overview). The effect was examined in several studies using a bar transport task (e.g., Rosenbaum et al., 1990). The task involves a horizontally oriented wooden dowel, raised high enough above a table to allow participants to grasp the dowel with an overhand (pronated/palm-down hand posture) or a more awkward underhand initial grasp (supinated/palm-up hand posture) to subsequently place the dowel onto a target. The studies consistently showed participants using initially awkward hand postures for the sake of a less awkward posture at the end of the transfer task. For example, when participants, planned to place the right end of the dowel onto a target using their right hand, they grasped the dowel with an overhand grasp, but when planning to place the left end of the dowel onto a target (also using the right hand), participants grasped the dowel with a rather uncomfortable underhand grip. In both cases the initially selected grip led to a more comfortable thumb-up (rather than down) end-state when the dowel was placed on the target. Action planning based on end-state comfort is often in effect in activities of daily living. For example, when grasping an object to subsequently use it, we typically apply a certain functional grip. *Functional grasping* describes the act of grasping a tool in a way that allows for its proper use (Creem and Proffitt, 2001; Randerath et al., 2009; Przybylski and Króliczak, 2017). When using a hammer to pound a nail, its handle must be grasped with the thumb pointing toward the hammer's head to allow for proper use. Alternatively, a less functional and biomechanically uncomfortable arm orientation, characterized by extreme joint angles would need to be adopted, to enable goal-directed (but extremely inefficient) use.

The ability to plan, select and execute grasps develops over time. For instance, the proportion of children between the ages of three and five who utilize underhand grips to achieve end-state comfort increases by approximately 25% with each increasing age group (Weigelt and Schack, 2010) and has been shown to increase in children up to the age of nine (Knudsen et al., 2012; Stöckel et al., 2012). However, these cohort effects may be bar rotation specific since planning abilities in other grasping tasks may become apparent at an even earlier age of 5 years (Jovanovic and Schwarzer, 2017; Herbort et al., 2018). It is certain however, that the ability can also be lost. A frequent functional deficiency after left middle cerebral artery stroke is limb apraxia (Buxbaum et al., 2007; Weiss et al., 2008), which affects motor cognition and is typically associated with impaired imitation of gestures or inappropriate pantomiming or actual handling of tools and objects (e.g., Randerath et al., 2011; Buxbaum et al., 2014; Goldenberg and Randerath, 2015; Weiss et al., 2016; Buchmann and Randerath, 2017). When handling tools, not only the use-movement can be affected. Difficulties in forward-planning can also be apparent in the preceding inappropriate non-functional grasping (Randerath et al., 2009, 2010). However, appropriate initial grasping may facilitate the subsequent production of effective object-use.

A helpful compensatory approach for patient populations suffering from impaired plan-based action-selection may be to use alternative routes that are rule-based. Rule-based actions are typically based on stimulus-response associations. We make use of them every day, e.g., when confronted with a red traffic light, we apply the brakes. When the light turns green, we accelerate. In social psychology and motivation science, if-then rules have been found to be effective in the context of *implementation intentions* (Gollwitzer, 1999). They have been shown to reduce cognitive demand by automating stimulus-response associations, thus facilitating goal-directed behavior (for a meta-analysis see Gollwitzer and Sheeran, 2006; for a review see Wieber et al., 2015). A recent meta-analysis demonstrated that if-then planning is also effective in clinical samples of patients with mental health problems (Toli et al., 2016) and recent studies have demonstrated implementation intention effects in physical endurance tasks (Bieleke and Wolff, 2017; Thürmer et al., 2017). Thus for patients with difficulties in forward planning, an alternative could be learning a simple rule that leads to an appropriate functional grasp (e.g., *if* I want to take a hammer, *then* I always grasp it with my thumb pointing toward the head).

To examine the applicability of such an idea to the domain of motor cognition, it is crucial to first systematically investigate the speed and accuracy of rule- compared to plan-based action-selection. One approach to dissociate plan-based from rule-based action-selection, while keeping factors such as stimuli and movement output similar is the rule/plan motor cognition (RPMC) paradigm (Randerath et al., 2013, 2015, 2017). In this paradigm, participants select pronated or supinated grasps and produce manual (object) rotation actions. In the rule-based task, the relationship between stimuli and grip type is fixed by instructed if-then rules. In the plan task, the relationship between stimuli and responses is flexible in that participants make use of a self-selected plan, based on end-state comfort. Results thus far have demonstrated faster reaction times in rule- vs. plan-based grip selection. Randerath et al. (2013) suggested that implementing rule-based grip selection leads to a reduction in cognitive workload, which is in line with the motivation literature on implementation intentions (e.g., Stewart and Payne, 2008; Janczyk et al., 2015). But see McCarty et al. (1999) or Herbort et al. (2017) for a different interpretation of the processing mechanisms underlying the end-state comfort effect.

In motor cognitive tasks, the robustness of these efficiency effects under different conditions remains unclear. For instance, pantomime actions are frequently preferred over real actions for study design because the experimental setting is easier to implement. Accordingly, previous implementations of the paradigm have used either pantomimed rotational movements (Randerath et al., 2015), or pantomimed grasping of familiar tools for which stimuli were presented via two-dimensional pictures (Randerath et al., 2013). Thus far, the applicability of previous (pantomime) results to actual object manipulation has not been tested and cannot be taken as self-evident, since action-mode may modulate efficiency effects. While similar action concepts may be retrieved, differences between modes could occur due to potential deviations in the demands on imagery, perception, on-line visuomotor control, and precision. For example, when grasping an object, pantomimed movements take longer compared to real movements, but object properties such as weight or size are taken into account in both action-modes (e.g., Goodale et al., 1994; Ansuini et al., 2016). For functional tool use, such as scooping soup, the action-modes differ in geometry and kinematics, but correlations of performance measures across the action-modes indicate that individual patterns are stable (Hermsdörfer et al., 2012, 2013). The delivered contextual information differs significantly between action-modes, whereby the level of affordances going along with the required action is manipulated. Compared to pantomime, an actual tool use setting provides fewer degrees of freedom for the required action, which may facilitate the planning process. In line with this, conceptual errors appear to be reduced when patients with tool use apraxia are confronted with a defined tool use setting (Randerath et al., 2011).

Further, accumulated evidence from research with neurological patients (for a review see Goldenberg, 2017) and neuroimaging studies have shown differences between pantomimed and real tool use execution on a neural level. For example, Króliczak et al. (2007) who used functional magnetic resonance imaging to compare the neural mechanisms of pantomimed and real grasping, showed that blood oxygenation level-dependent signal strength significantly differed between real reaching and real grasping, but not between pantomimed reaching and pantomimed grasping.

Here, the main goal was to examine whether the efficiency effects previously found in the RPMC paradigm remain stable across the mode of execution. We propose that compared to end-state comfort based planning, applying an implementation intention based rule expedites action initiation for pantomimed as well as real movements. However, the extent of such efficiency effects may be modulated by the action-mode. The mode producing higher workload (pantomime) is expected to produce relatively stronger efficiency effects. To test this hypothesis, an automated apparatus capable of measuring rotation times and grip orientation was built and applied. It should be noted, that different from typical end-state comfort tasks as described above, we explicitly instructed participants to grasp in a comfortable way.

**Figure 1:**
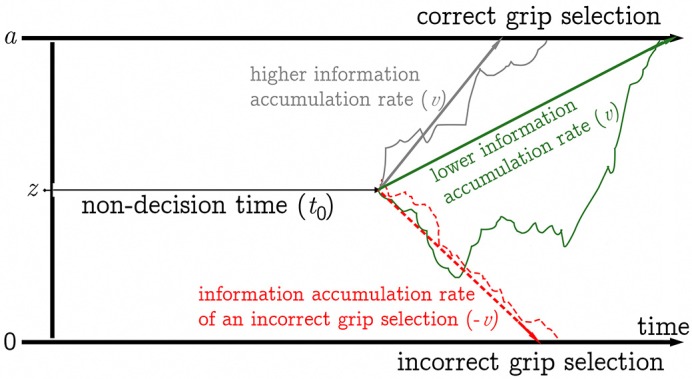
Schematic illustration of the diffusion model. Non-decision time components are combined in the *t*_0_ parameter (represented by the thin black arrow). Three decision processes are shown (solid paths lead to correct grip selection, dashed path leads to incorrect grip selection). The decision processes start between the two decision boundaries (at 0 and *a*) at the level of the starting point *z*. A grip is selected when the corresponding decision boundary is reached. The gray path shows a decision process with a relatively higher mean rate of information accumulation (high drift rate (*v*), represented by the gray arrow), the green path shows a decision process with a lower mean rate of information accumulation (lower *v*, represented by the green arrow). The red path shows a decision process leading to an incorrect grip selection, the red arrow represents the corresponding rate of information accumulation.

As we are particularly interested in the information processing component of reaction times (grip selection), which can be masked by differences in speed-accuracy trade-off, speed of motor-response encoding and other processes occurring between stimulus presentation and response, we ran a simulation study to show the suitability of our collected reaction time data for drift diffusion modeling (DDM; Voss et al., 2013, 2015), and describe the effects of our experimental manipulations on the correct selection of pronated or supinated grip postures in terms of diffusion model parameters. The Ratcliff diffusion model (Ratcliff, 1978; Ratcliff and McKoon, 2008) represents the decision process as a Wiener process originating from a starting point (*z*) located between two decision boundaries (commonly an upper boundary *a* and a lower boundary 0). A decision is made when one of the two decision boundaries is reached. In other words, decisions are modeled as noisy stochastic processes that drift toward decision boundaries as information accumulates. In binary decision tasks (i.e., selection of pronated vs. supinated posture) the diffusion model allows for the statistical decomposition of reaction times into parameters reflecting (among others) the rate of information accumulation (drift rate, *v*), distance between decision thresholds (boundary separation, *a*), and duration of non-decision components (e.g., stimulus encoding, preparation of motor response, task switching, visualization) combined in the non-decision time parameter *t*_0_ or *T*_*er*_ (Voss et al., 2004). In a diffusion model with an unbiased starting point, the reaction time difference between a given relatively slower decision (hypothetically in the plan-based task) and a relatively faster decision (hypothetically in the rule-based task) can be accounted for by differences in *a*, *v*, *t*_0_ or a combination of those parameters. Lower *a* indicates that less information is required for a decision to be made, leading to faster reaction times and a higher error probability (more liberal response criterion). Lower *v* implies less efficient processing of information. Differences in *t*_0_ between conditions indicate that processes not directly involved in the decision differ (see Figure 1).

In *Experiment A* we directly compare pantomime and real object implementations of the RPMC paradigm in a within-subjects design. Similar to previous results in studies applying the RPMC paradigm with pantomimed actions (Randerath et al., 2013, 2015, 2017), we predict that reaction times will be faster in rule trials than in plan trials. We expect the drift parameter *v* to be larger in the rule task, as the strengthening of the stimulus-action link suggested by the implementation intention literature should increase the efficiency of information uptake. Furthermore, we expect faster reaction times for the more typical overhand grasp than for underhand grasps, and equally fast reaction times for the dominant and non-dominant hands, in line with previous research by Randerath et al. (2013).

As the pantomime condition places greater demand on mental imagery, this increase in cognitive workload is proposed to increase *t*_0_. Furthermore, we hypothesize that relative differences in reaction times between rule and plan tasks will be larger in the pantomime-movement condition compared to the real object condition, as rule-based action-selection should allow greater allocation of cognitive resources toward the more demanding motor imagery processes involved in the pantomime condition.

In *Experiment B* we investigate the effects of task switching in the real object manipulation action-mode by comparing two versions of the experiment, within-subjects. In the mixed version, rule- and plan-based actions were presented in a pseudo-randomly mixed sequence, while the blocked version consisted of a rule-task-only and a plan-task-only block. As in Experiment A, we predict that reaction times will be faster in rule trials than in plan trials and expect the rate of information uptake, *v*, to be larger in the rule task than in the plan task. Also as in Experiment A, we expect faster reaction times for the more typical overhand grasp than for underhand grasps, and equally fast reaction times for the dominant and non-dominant hands. In the mixed condition, prolonged reaction times compared to the blocked condition are predicted because of increased cognitive load attributable to task switching costs (Monsell, 2003). As such, we expect *t*_0_ to be higher in the mixed condition than in the blocked condition (Schmitz and Voss, 2012, 2014). As the presentation of trials in task-pure compared to mixed fashion should lead to an increase in task readiness, we expect a higher drift rate *v* in the blocked condition compared to the mixed condition (Schmitz and Voss, 2012, 2014).

Moreover, the experiments aim to elucidate the extent of the similarity of motor outputs elicited by either plan- or rule-based action planning by measuring the duration of handle rotations. Thus far, there has been no attempt to extract the rotation component in the context of the RPMC paradigm.

To summarize, the aim of this work is to examine the behavioral stability of efficiency effects in the RPMC paradigm in different action-modes and under conditions of varying cognitive load. To complement typical RT analyses (ANOVA), we applied diffusion modeling as it seems to be a suitable approach for the analysis of action selection processes within the present paradigm.

## 2. Experiment A

### 2.1. Methods

Experiments A and B were approved by the ethics committee of the University of Konstanz. All participants gave written informed consent in accordance with the Declaration of Helsinki.

#### 2.1.1. Participants

Based on the task means reported in Randerath et al. (2015; Experiment 3 (*N* = 21); condition averages, Plan: *M* = 966.5 ms, *SD* = 154.4 ms, Rule: *M* = 801.2 ms, *SD* = 96.1) we calculated a minimum sample size of *N* = 8 (with α = 0.05, *power* = 0.8) using G*Power 3 (Faul et al., 2007), to detect task differences in pantomime condition RTs. For this experiment we included a sample of 16 healthy participants, since we hypothesized that task effects would be reduced in the real action-mode. The sample consisted of 12 female and 4 male participants with a mean age of 25.4 years (*SD* = 4.7 years). All participants had either recently received or were currently pursuing a university degree. Handedness was assessed using the Edinburgh Handedness Inventory version by Salmaso and Longoni (1985). One participant was left-hand dominant; all other participants were right-hand dominant. Participants received either study-credits or 20 EUR for their participation and were assigned to one of four conditions to counterbalance the order of real and pantomime sessions and task-cue color assignment (see next section). The assignment was matched for age. Instructions were given in German. The experimenter confirmed language fluency. Participants had normal or corrected-to-normal vision.

#### 2.1.2. Materials and procedure

Each participant was tested in two 45–60 min sessions, namely, a real object session and a pantomime session. These sessions were no more than 3 days apart. All participants gave written consent to both participation and video recording of the experimental sessions.

The experiment was presented with SuperLab 5 (Cedrus Corporation, San Pedro, CA, USA) on a 24-inch screen at a resolution of 1,920 × 1,080 pixels, run from on-board graphics of an Intel Core i7 4790 @3.6 GHz CPU with 16GB of RAM running a 64-bit version of Windows 8.1.

**Figure 2:**
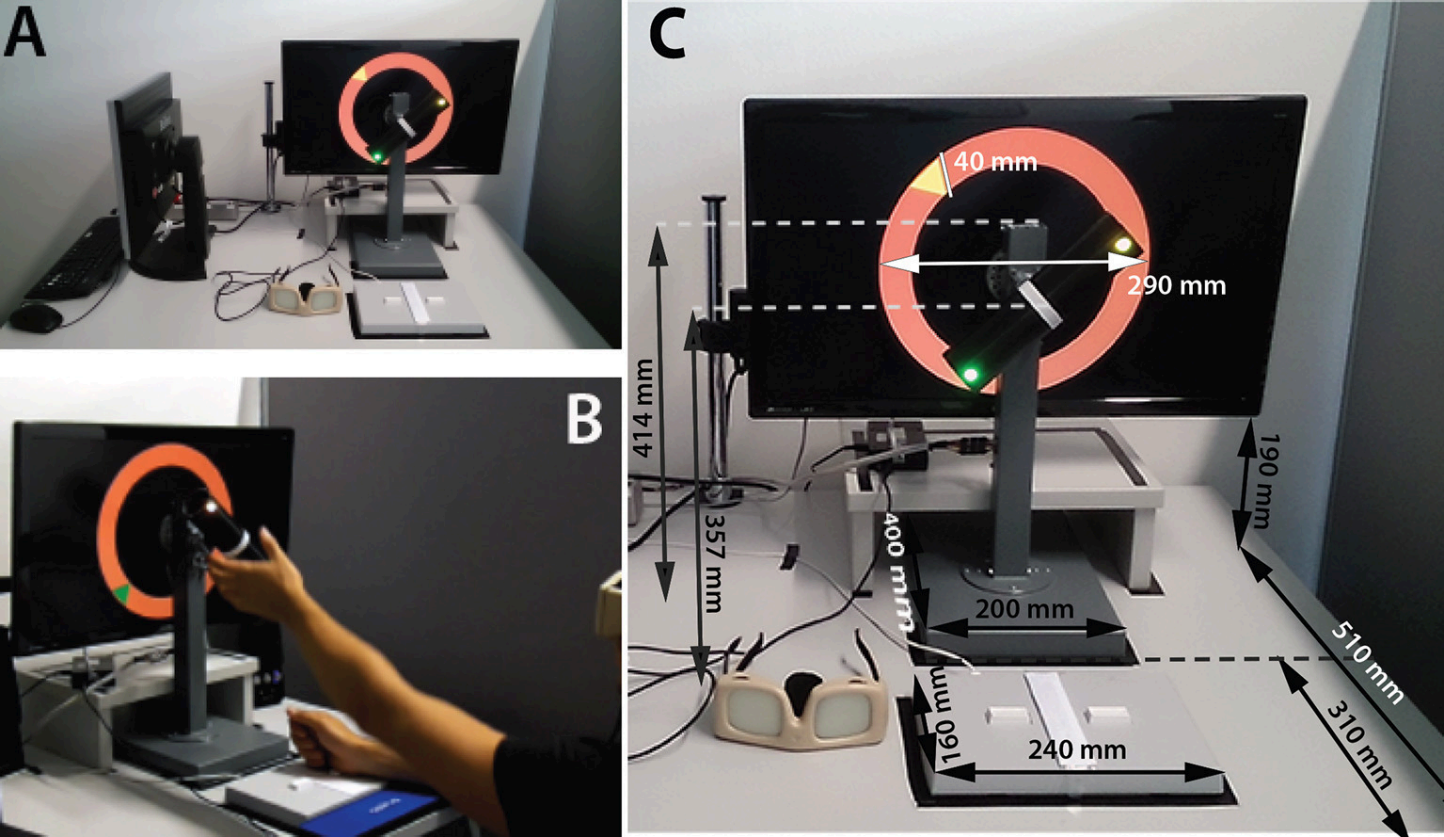
**(A)** shows the experimental setup (without speakers) with the monitor showing an underhand trial for the right hand. The visual occlusion goggles in their closed state are located to the left of the two-button response pad. The experimenter monitor with keyboard and mouse, used to input participant data, on-line grip error coding, and general experiment control can be seen on the left of the image. **(B)** shows a participant executing an underhand grasp with the left hand, while using the inactive right hand to keep the right response button pressed as instructed. **(C)** shows dimensions of the equipment used and distances between the equipment. Arrows leaving the picture plane extend to the forward edge of the table (80 mm from the forward edge of the response pad). Distance vectors parallel to the table plane are parallel and perpendicular to respective table edges. Vertical distance vectors are orthogonal to the table plane.

The experimental setup (see Figure 2) was adjusted to place the center of the screen's viewable area at participants' eye level, with chair height adjusted to put participants' thighs and shins at a 90° angle with feet flat on the floor. This was accomplished by placing the experimental setup on a height-adjustable table, which allowed the distances between the monitor, RPMC apparatus (see Figure S1 for details), and response pad to be kept identical for all participants while keeping viewing angles constant (see Figure 2C).

Participants wore Translucent Technologies PLATO visual occlusion goggles (http://www.translucent.ca), which hid the monitor and apparatus from view before each trial. Participants were instructed to keep both buttons of the response pad (Lumina RB-540, Cedrus corporation) pressed with a loosely balled fist (thumbs away from the response buttons, 5th fingers toward the response buttons) while the goggles were “shut” (lenses switched to their opaque state). Before each trial, a voice recording (1,000 ms duration) saying either “left hand” or “right hand” instructed participant which hand to use for the upcoming trial. The recording was followed by a variable inter-stimulus-interval (500, 800, or 1,100 ms). As soon as the goggles “opened” (lenses switched to their transparent state) participants had to release the respective response button, perform the handle rotation task, and then return the hand to the response button, which triggered opacity of the lenses before the next trial (see Figures 2A,C). Reaction times were measured as the interval between the opening of the goggles and button release. Movement times measured the interval between button release and button press.

The implementation of the RPMC paradigm used in Experiments A and B consisted of 128 trials (8 × 16 trial types) per session. The 16 trial types were based on: 2 tasks (plan vs. rule) × 2 grips (overhand vs. underhand) × 2 hands (left vs. right) × 2 colors of the target (i.e., green or yellow vs. blue or magenta). Light emitting diodes (LEDs) on the ends of the apparatus handle (Figure 2) specified the task (rule or plan). LED color combinations (e.g., Plan: green/yellow, Rule: blue/magenta) were counterbalanced between participants. Trials further varied with respect to LED color placement (i.e., green on the right or left side of the handle etc.) and on-screen location of the target (upper vs. lower and right vs. left quadrant of the on-screen circle), see Figure 2. Initial handle angles (from the participant perspective) were 45° for the right hand and 315° for the left hand, relative to the vertical (0° or “12 o'clock”) position (see Figure 2A). Those angles were selected because previous research by Johnson (2000) has shown hand orientations of those angles to be rated as equally comfortable. Successful handle rotation always spanned an angle of 90°.

The 128 trials per session were divided into four blocks of 32 trials. In each of the four blocks the 16 trial types were presented twice. Trials in each block were presented in pseudo-randomized order, such that there would not be more than three consecutive trials which utilized the same hand, task, or grip.

Participants were instructed to rotate the handle in a way that would align the colored arrow (40 mm equilateral triangle) with the light on the handle of the same color as the arrow and that the apparatus would block further rotation when they had rotated the handle far enough. Participants were further instructed to execute each rotation as comfortably as possible and that this meant that hand posture before and after the rotation should be comfortable and natural. Participants were shown an example of a comfortable or an uncomfortable end position. This instruction was given independently of task. They were further told that the middle finger should remain in contact with a white strip of tape affixed to the center of the handle, which spanned the visible length of the handle's equator. It was also emphasized, that thumb and index fingers should be on the same side of the white strip, regardless of hand posture. It was then explained that the white strip divided the handle into two sides, meaning the “green side” was the side on which the green light was, and the “yellow side” was the side on which the yellow light was and so on. Participants were explicitly told to rotate the handle in the direction that constituted the shortest distance.

Participants were then informed that they would be completing two different tasks, referred to as the green/yellow and the pink/blue (most participants referred to magenta as “pink”) tasks. For half of the participants the green/yellow task was the plan task; for the other half, it was the rule task. For the sake of simplicity, the following task description will assume a participant for whom green/yellow coded for the rule task, and pink/blue coded for the plan:

In the plan task participants were instructed to rotate the pink light to align with the pink arrow or the blue light to align with the blue arrow. To do so, participants were asked to form the intention to execute the movement as comfortably as possible. They were then presented with a cardboard sheet showing two rectangles of pink and blue color, with a text stating “*I will execute the movement as comfortably as possible*.” printed on it, and were asked to read the statement out loud.

For the rule task, participants were told to grasp the handle such that their thumb would be on the same side of the handle as the light of the same color as the arrow stimulus. That instruction was repeated once before participants were instructed to form the following implementation intentions: “*If the arrow is green, then I will place my thumb on the green side of the handle*” and “*If the arrow is yellow, then I will place my thumb on the yellow side of the handle*.” Participants were then shown another piece of cardboard, this time showing green and yellow rectangles, displaying the text of the intentions they were asked to form for the rule task. Participants then read the printed text out loud. They then completed 16 practice trials (which included all stimulus/handle combinations). Both pieces of cardboard remained visible to participants to the left and right of the PC monitor for the duration of the 16 RPMC training trials.

Participants were tested in two sessions. In Experiment A half of the participants received the pantomime version of the RPMC experiment in the first session, while the other half received the real object version first. Color combinations indicating plan or rule trials were kept constant across sessions for each participant. Prior to the start of the RPMC paradigm participants were instructed to perform the task as quickly as possible but with an emphasis on accurate task performance.

#### 2.1.3. Data analysis

Dependent variables in the RPMC paradigm were reaction time (RT, time from opened goggles to button release), movement time (MT, time between button release and button press), and errors. Additionally, handle rotation time (rotTime) was measured by the apparatus. For technical reasons (see supplement) measurement of rotation time began as soon as the handle deviated greater than 3.125° from the start position in the proper direction of rotation (remaining span of handle rotation at *T*_0_ = 86.875°).

For each participant, the data were stratified by combinations of action-mode (real or pantomime), task (rule or plan), grip (pronated or supinated) and hand (non-dominant or dominant). Trials containing erroneous participant responses in the RPMC task were identified from records of on-line participant observation and confirmed by review of recorded video material. Errors were coded when participants utilized either the wrong hand or grip type (which would lead to an uncomfortable end position), rotated the handle in the wrong direction, or removed their hand from the response pad before the PLATO goggles opened. These trials were removed from the data set prior to outlier screening. Error trials, as well as trials containing time measure outliers, were excluded from the analysis of reaction and movement times. The Generalized Extreme Studentized Deviate (ESD) test for multiple outliers (Rosner, 1983) was used to detect outliers. Normality of time-measure residuals was assessed by reviewing normal probability plots and with the Kolmogorov-Smirnov test which indicated that RT, MT, and rotation time residuals were approximately normally distributed, *p* > 0.15.

We calculated three repeated measures analyses of variance (ANOVA) using Statistica Version 13 (http://software.dell.com); one for each of the dependent variables RT, MT, and rotation time. Based on our hypotheses, each ANOVA was constrained to the main effects of task (rule/plan), grip (pronated/supinated), hand (non-dominant/dominant), and action-mode (pantomime/real) as well as the task^*^mode interaction. We calculated *t*-contrasts comparing tasks in each action-mode to test our RT hypothesis. To correct for family-wise error rate, we adjusted *p*-values using the Holm-Bonferroni procedure. Significant interactions in variables other than RT were analyzed with Bonferroni *post-hoc* tests, since we only formulated a priori hypotheses for RTs.

For the analysis of grip-error data, we used non-parametric tests as the data were not normally distributed (Kolmogorov-Smirnov test: *p* < 0.05). The number of grip errors (utilizing pronated grips in trials requiring a supinated grip and vice versa) was compared using the Wilcoxon-Test for paired samples in four sets of comparisons (Plan vs. Rule trials, Overhand vs. Underhand trials, Dominant vs. Non-dominant hand trials, Real vs. Pantomime trials) at a Bonferroni corrected α level of α_bf_ = 0.0125 to account for multiple comparisons.

##### 2.1.3.1. Diffusion model analysis and simulation study

Diffusion model parameters were estimated using the fast-dm-30.2 program (Voss and Voss, 2007, 2008; Voss et al., 2015). Accuracy-coded data-sets for diffusion model analysis were created for each participant by adding the previously removed grip-errors to the outlier free data sets. Additionally, an upper cut-off of 1800 ms and a lower cut-off of 200 ms was applied to error RTs. At the lower cut-off, on average, correctness of grip selection was approximately at chance-level (Ratcliff and McKoon, 2008). Following the recommendation of Ratcliff and Tuerlinckx (2002) the upper cut-off was set so that 0.5% of responses were slower than the upper cut-off. The parameters *v, t*_0_, and *a* were allowed to vary by all possible combinations of action-mode and task, but not grip and hand to increase the number of trials in each factor combination and thus improve the reliability of parameter estimates. The parameter *d*, which represents the difference in the speed of response execution between correct and incorrect responses was allowed to vary by participant. To counteract the negative effect of possible fast contaminant RTs, the inter-trial non-decision time variability parameter *st*_0_ was added and allowed to vary in the same way as *v, t*_0_, and *a* (Lerche and Voss, 2016). Since the data were accuracy coded, the starting point *z* was fixed to 0.5 (no response bias). All other parameters were fixed to 0. Given the relatively low number of trials, Maximum-Likelihood was chosen as the optimization criterion. For further details see Voss et al. (2015).

To confirm that the RPMC paradigm is apt for diffusion modeling we ran a simulation study following the recommendations in Voss et al. (2015). In each experiment, 5,000 data sets per participant were simulated by first creating multivariate normal distributions defined by the covariance matrix of empirical parameter estimates (MATLAB script can be downloaded from https://github.com/MoCogKonstanz/RPMC) from which simulated parameter estimates were drawn. The simulated parameter estimates were then split by condition and participant. Subsequently, the *construct-samples* tool included in fast-dm was used to create 5,000 data sets for each participant in each of the four factor combinations with 64 trials each. These condition-specific data sets were then merged into 5,000 complete data sets per participant. In turn, the 5,000 complete data sets were entered into diffusion model analysis using the same settings as for the analysis of empirical data. For each simulated data set, the 95th percentile of fit indices was calculated. Following the recommendations of Voss et al. (2015), all empirical data sets fitting worse than 5% of the worst fitting corresponding simulated data sets (>95th percentile) would have been excluded from diffusion model analysis, as fits worse than the 5% criterion would have indicated that those data sets are not suitable for diffusion modeling. As all empirical data sets fit the diffusion model better than the 5% criterion suggested by Voss et al. (2015), we conclude that the paradigm is suitable for diffusion modeling. Fit indices for Experiment A and Experiment B data sets are shown in Table S1.

### 2.2. Results experiment A

#### 2.2.1. Reaction time

**Figure 3:**
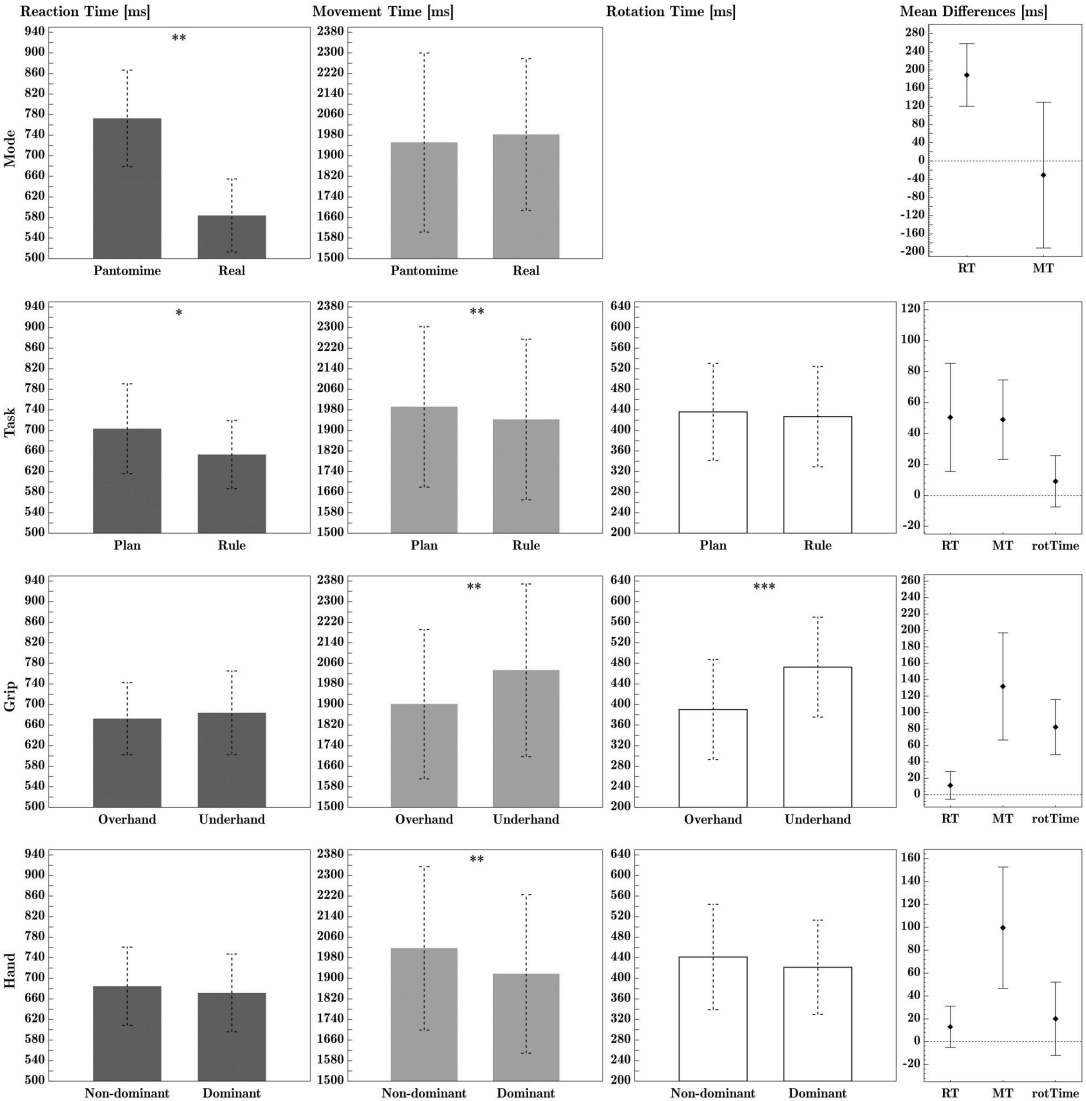
Experiment A ANOVA main effects, for (from left to right column) reaction time, movement time and rotation time. Within-factor differences are shown in the far right column. All times are given in milliseconds. Error bars represent 95% confidence intervals. ^*^, ^**^, and ^***^ denote significant differences with Holm-Bonferroni adjusted *p*-values *p*_*adj*_ < 0.05, < 0.01, and < 0.001, respectively. Calculation of mean difference times: Mode difference = (Pantomime − Real); Task difference = (Plan − Rule); Grip difference = (Underhand − Overhand); Hand difference = (Non-dominant − Dominant). See Table 3 for effect sizes and *p*-values.

The ANOVA of RTs showed a significant main effect of action-mode, with slower mean RTs in the pantomime session of the experiment than in the real object session. There was also a main effect of task, with participants showing slower mean RTs in the plan task than in the rule task. There was a significant interaction between task and action-mode (see **Figure 5**). The planned comparisons showed that rule RTs were significantly faster than plan RTs in the pantomime action-mode, *t*_(15)_ = 3.32, *p* = 0.005, but not in the real action-mode, *t*_(15)_ = 1.27, *p* = 0.224. There were no significant main effects of grip or hand on RTs. See Tables 1, 2 for main effect and interaction means, respectively. See Table 3 for comprehensive ANOVA results. Main effects are shown in Figure 3. Full factorial data are given in Tables S2, S3.

**Table 1:** Main effect means and standard deviations.

**Experiment**	**Factor**	**Level**	**RT**		**MT**		**rotTime**	
			***M***	***SD***	***M***	***SD***	***M***	***SD***
Exp. A	Mode	Pantomime	772.6	170.9	1951.5	632.1		
		Real	583.8	135.6	1982.6	543.6		
	Task	Plan	703.4	159.3	1991.6	569.5	436.1	173.0
		Rule	653.0	126.4	1942.6	571.8	426.9	176.9
	Grip	OH	672.5	130.5	1901.1	533.2	390.3	176.5
		UH	683.8	150.6	2033.0	611.0	472.6	177.8
	Hand	ND	684.7	143.6	2016.9	583.0	441.5	185.3
		Dom	671.7	138.5	1917.3	561.4	421.4	168.1
Exp. B	Condition	Blocked	620.8	100.0	1701.9	234.7	349.4	66.7
		Mixed	712.8	142.3	1798.8	300.1	339.7	96.5
	Task	Plan	691.2	118.8	1773.7	222.4	345.5	77.3
		Rule	642.4	112.6	1727.1	255.5	343.6	76.4
	Grip	OH	654.5	103.6	1656.7	231.7	310.7	72.1
		UH	679.1	125.5	1844.1	240.7	378.3	78.0
	Hand	ND	670.4	112.9	1767.0	229.2	337.5	76.4
		Dom	663.2	116.1	1733.7	237.1	351.6	75.0

*The table gives means (M) and standard deviations (SD) of Experiment A and Experiment B main effect reaction times (RT), movement times (MT), and rotation times (rotTime) in milliseconds. ND, non-dominant hand; Dom, dominant hand; OH, overhand grip; UH, underhand grip*.

**Table 2:** Interaction means and standard deviations.

**Experiment**	**Session**	**Task**	**RT**		**MT**		**rotTime**	
			***M***	***SD***	***M***	***SD***	***M***	***SD***
Exp. A	Pantomime	Plan	816.3	210.7	1988.3	618.8		
		Rule	728.8	140.0	1914.8	648.7		
	Real	Plan	590.4	136.3	1994.8	555.8	436.1	173.0
		Rule	577.1	138.1	1970.4	534.1	426.9	176.9
Exp. B	Blocked	Plan	642.5	132.5	1701.4	307.1	345.3	93.9
		Rule	599.1	127.0	1702.5	300.0	353.5	86.1
	Mixed	Plan	739.8	187.9	1846.0	351.1	345.7	115.7
		Rule	685.7	165.6	1751.6	395.3	333.6	117.9

*The table gives means (M) and standard deviations (SD) of Experiment A and Experiment B interaction effect reaction time (RT), movement time (MT), and rotation time (rotTime) data in milliseconds*.

**Table 3:** Experiment A and B ANOVA results.

**Experiment**	**Effect**	**RT**				**MT**				**rotTime**			
		***F***_(1, 15)_	***p***	***p***_**adj**_	$\eta_{p}^{2}$	***F***_(1, 15)_	***p***	***p***_**adj**_	$\eta_{p}^{2}$	***F***_(1, 15)_	***p***	***p***_**adj**_	$\eta_{p}^{2}$
Exp. A	Intercept	375.27	0.000		0.96	190.47	0.000		0.93	98.05	0.000		0.87
	Mode	33.99	0.000	0.000^***^	0.69	0.17	0.684	−	0.01				
	Task	9.51	0.008	0.023^*^	0.39	16.60	0.001	0.004^**^	0.53	1.39	0.257	−	0.08
	Grip	2.09	0.169	−	0.12	18.57	0.001	0.003^**^	0.55	27.41	0.000	0.000^***^	0.65
	Hand	2.35	0.146	0.293	0.14	16.05	0.001	0.003^**^	0.52	1.77	0.203	0.406	0.11
	Mode^*^Task	10.12	0.006	0.025^*^	0.40	1.80	0.200	0.400	0.11				
		***F***_**(1, 23)**_	***p***	***p***_*adj*_	$\eta_{p}^{2}$	***F***_(1, 23)_	***p***	***p***_*adj*_	$\eta_{p}^{2}$	***F***_**(1, 22)**_	***p***	***p***_*adj*_	$\eta_{p}^{2}$
Exp. B	Intercept	549.71	0.000		0.96	910.48	0.000		0.98	347.86	0.000		0.94
	Condition	15.52	0.001	0.003^**^	0.40	2.00	0.170	−	0.08	0.27	0.609	−	0.02
	Task	20.77	0.000	0.001^***^	0.47	2.47	0.130	−	0.10	0.03	0.858	−	0.01
	Grip	8.26	0.009	0.026^*^	0.26	71.42	0.000	0.000^***^	0.76	99.42	0.000	0.000^***^	0.83
	Hand	1.15	0.295	−	0.05	8.39	0.008	0.033^*^	0.27	2.98	0.099	−	0.14
	Condition^*^Task	0.24	0.632	−	0.01	7.55	0.011	0.034^*^	0.25	1.71	0.204	−	0.04

* *The table gives effect sizes and significance of Experiment A and B repeated measures ANOVAs. Effects with Holm-Bonferroni adjusted p-values p_adj_ < 0.05 are marked*,

** *with p_adj_ < 0.01 are marked*,

*** *with p_adj_ < 0.001 are marked*.

*Effect sizes are reported as partial eta squared ($\eta_{p}^{2}$) values. RT, reaction time; MT, movement time; rotTime, rotation time*.

##### 2.2.1.1. Diffusion parameters

Action-mode had a significant effect on *t*_0_ with significantly longer non-decision times in the pantomime action-mode than in the real action-mode. Task had a significant main effect on drift rate, with significantly higher *v* in the rule task compared to the plan task. The mode^*^task interaction of drift rate did not survive family-wise error correction. The main-effect of Task on boundary separation (*a*) was not significant after family-wise error correction. The significant mode^*^task interaction showed significantly lower *a* for the plan task compared to rule task only in the pantomime action-mode, *p* = 0.006. See Table 5 for DDM ANOVA results and Table 4 for DDM means and standard deviations. See **Figure 6** for mode^*^task interaction plots of diffusion model parameters.

**Table 4:** Experiment A and B diffusion model means and standard deviations.

**Experiment**	**Condition**	**Task**	**Mean v**	**SD *v***	**Mean *t*_0_**	**SD *t*_0_**	**Mean *a***	**SD *a***
Exp. A	Pantomime	Plan	3.09	1.26	0.580	0.149	1.41	0.59
		Rule	5.93	2.63	0.515	0.162	2.36	0.97
	Real	Plan	4.73	2.11	0.427	0.137	1.49	0.64
		Rule	5.65	2.57	0.433	0.142	1.50	0.79
Exp. B	Blocked	Plan	4.43	1.77	0.485	0.10	1.70	0.93
		Rule	6.19	2.16	0.430	0.13	2.39	1.31
	Mixed	Plan	3.62	2.40	0.517	0.14	1.93	1.53
		Rule	4.56	2.30	0.492	0.15	2.14	1.53

*The table gives means and standard deviations of Experiment A and B drift parameters. t_0_ is given in seconds. v, drift rate; t_0_, non-decision time; a, boundary separation*.

**Table 5:** Experiment A and B diffusion model parameter ANOVA results.

**Experiment**	**Effect**	***v***				***t***_**0**_				***a***			
		***F***_(1, 15)_	***p***	***p***_**adj**_	$\eta_{p}^{2}$	***F***_(1, 15)_	***p***	***p***_**adj**_	$\eta_{p}^{2}$	***F***_(1, 15)_	***p***	***p***_**adj**_	$\eta_{p}^{2}$
Exp. A	Intercept	180.18	0.000		0.92	222.96	0.000		0.94	316.88	0.000		0.96
	Mode	1.44	0.249	–	0.09	30.97	0.000	0.000^***^	0.67	3.47	0.082	–	0.44
	Task	18.27	0.001	0.002^**^	0.55	3.29	0.090	0.179	0.18	6.09	0.026	0.052	0.09
	Mode^*^Task	4.85	0.044	0.087	0.24	2.54	0.132	–	0.14	8.08	0.012	0.037^*^	0.24
	
		***F***_(1, 23)_	***p***	***p***_**adj**_	$\eta_{p}^{2}$	***F***_(1, 23)_	***p***	***p***_**adj**_	$\eta_{p}^{2}$	***F***_(1, 23)_	***p***	***p***_**adj**_	$\eta_{p}^{2}$
Exp. B	Intercept	254.18	0.000	0.92	535.51	0.000	0.96	190.12	0.000	0.96			
	Condition	11.24	0.003	0.008^**^	0.33	4.84	0.038	0.114	0.17	0.00	0.978	−	0.16
	Task	9.37	0.006	0.011^*^	0.29	4.17	0.053	−	0.15	2.38	0.136	0.273	0.05
	Cond^*^Task	1.48	0.235	0.235	0.06	0.59	0.450	−	0.03	0.74	0.400	−	0.14

* *The table gives effect size and significance for Experiment A and B repeated measures ANOVAs of the drift parameters v, t_0_, and a. Effects with Holm-Bonferroni adjusted p-values p_adj_ < 0.05 are marked*,

** *with p_adj_ < 0.01 are marked*,

*** *with p_adj_ < 0.001 are marked*.

*Effect sizes are reported as partial eta squared ($\eta_{p}^{2}$) values. v, drift rate; t_0_, non-decision time; a, boundary separation*.

#### 2.2.2. Movement time

The ANOVA revealed a significant main effect of task on MTs, with faster MTs in the rule task than in the plan task. The significant effects of grip type showed faster MTs in overhand trials than in underhand trials. Dominant hand MTs were significantly faster than non-dominant hand movement times. See Tables 1, 2 for main effect and interaction means, respectively. See Table 3 for comprehensive ANOVA results. Main effects are shown in Figure 3. Full factorial data are given in Tables S2, S3.

#### 2.2.3. Rotation time

There was a significant main effect of grip on rotation times, with overhand grips producing faster rotation times than underhand grips. As rotation times were measured with the apparatus, the result applies only to the real object mode session. See Tables 1, 2 for main effect and interaction means, respectively. See Table 3 for comprehensive ANOVA results. Main effects are shown in Figure 3. Full factorial data are given in Tables S2, S3.

#### 2.2.4. Grip errors

There were a total of 77 grip errors in 4096 trials. The mean error rate was 1.88%. There were 2048 trials in the plan task as well as in the rule task. In the plan task, 62 errors (3.027%) were observed, and in the rule task, 15 errors (0.732%) were observed. Moreover, of 2048 trials in the pantomimed-movement condition, 45 (2.197%) produced erroneous hand postures. In the real-movement condition, 32 errors (1.562%) were observed in 2048 trials. Pair-wise Wilcoxon comparisons of task showed that the number of grip errors significantly differed only in the plan, *M* = 3.88, *SD* = 3.24, vs. rule, *M* = 0.94, *SD* = 1.61, comparison, *W*(13) = 4.0, *Z* = 2.90, *p* = 0.004. The real, *M* = 2.00, *SD* = 1.93, vs. pantomime, *M* = 2.81, *SD* = 2.90, error comparison was not significant with, *W*(11) = 24.5, *Z* = 0.76, *p* = 0.45.

### 2.3. Discussion experiment A

In this experiment, the same actions could be achieved with either rule-based or plan-based instructions. In contrast to plan-based actions our rule-based actions were guided by implementation intentions (if-then rules), and as expected we found faster processing for this task compared to when prospective planning was involved. Our major point of interest was how action-mode modulates this processing advantage. We found that efficiency effects are stronger in the pantomime mode vs. when using the real object. Further, timing across the movement stages was affected differently by the assessed variables. Effect sizes ($\eta_{p}^{2}$) indicate that the movement initiation phase (RT) seems to be predominantly influenced by action-mode and task, while the time for hand transport (MT) is still modulated by task but also by grip type as well as the used hand. Rotation time appeared to be predominantly influenced by grip type.

#### 2.3.1. Task and action-mode effects

As hypothesized, rule task RTs were shorter than plan task RTs, particularly in the pantomime mode. The larger advantage of rule-based action-selection over plan-based action-selection in the pantomime compared to the real action-mode is illustrated by the significant interaction between task and action-mode (see **Figure 5**). Although we measured a slight advantage of rule RTs over plan RTs in the real object condition (on average 13.3 ms), this tendency was not statistically significant. Interestingly, MTs were affected by task similarly to RTs (see Figure 5). It is possible that a portion of the grip selection process carried over into the movement phase (measured by MTs) and thus reduced the task difference in the planning phase (measured by RTs). This likely affected the real action-mode proportionally stronger due to the smaller magnitude of the task difference.

As expected, drift rates appeared to be sensitive to task, with participants showing significantly higher drift rates (*v*) in the rule task than in the plan task. This confirms that the rule advantage, judging by effect sizes given in Table 5, primarily stems from an optimized grip-selection process, rather than optimization of decision boundaries or non-decision components. With regard to grip errors, the analyses show significantly more errors in the plan task than in the rule task. Though more grip errors were made in the pantomime-movement condition than in the real-movement condition, the difference between action-modes was not significant. However, in the pantomime condition, the diffusion model showed that participants adopted significantly wider decision boundaries (larger *a*) in the rule task than in the plan task, indicating more cautious responding. It appears that the higher rates of information accumulation in the rule task are able to offset this boundary separation induced latency, as RTs in the pantomime action-mode were significantly faster in the rule task than in the plan task. Finally, the prolonged non-decision time (*t*_0_) in the pantomime condition compared to the real condition suggests that differences in non-decision times (*t*_0_ parameter) between action-modes may be good approximations of the time required for movement imagery.

#### 2.3.2. Effects of hand and grip type

We found a dominant hand advantage for MTs. One likely explanation is that grasping movements are usually performed with the dominant hand and are thus more trained. Overhand grasps produced significantly shorter MTs and rotation times than underhand grasps. Similar to the dominant hand advantage in MTs, we interpret the overhand advantage in terms of training by way of more frequent use. We speculate that biomechanical constraints may have contributed to the longer rotation times in underhand grasps. Although the handle angles we used have previously been rated as equally comfortable for pronated and supinated hand postures, it is possible that overhand grips (during initial grasping, thumbs were pointed inward and down) induced more muscle tension in the forearm during initial grasping, which could have led to faster handle rotations.

#### 2.3.3. Experiment A conclusion

Succinctly, the planning phase seems to be predominantly influenced by action-mode and task. This resulted in slower responses in the pantomime condition compared to the real condition, and in line with the findings reported by Randerath et al. (2013, 2015, 2017), this also resulted in faster responses during rule-based action initiation compared to plan-based initiation. The execution phase appears to be modulated by hand, grip type, and task. Participants demonstrated faster movements when solving tasks in the real vs. the pantomime condition, quicker execution of overhand compared to underhand grasping movements, and faster movements in rule compared to plan trials. As hypothesized and previously reported by Randerath et al. (2013), there was no significant difference between dominant and non-dominant hands in the planning phase. However, in the movement phase, dominant hand movements were significantly faster than non-dominant hand movements. Contrary to our hypothesis and the findings of Randerath et al. (2013), we did not find a significant RT difference as a function of grip posture, in the planning phase. The extracted rotation-movement however, appears to be predominantly influenced by grip type, with overhand rotations being faster than underhand rotations. Here, task does not appear to have an effect.

To conclude, time measures of movement planning and movement execution are susceptible to efficiency effects, with quicker responses for parameters inducing less cognitive load or higher familiarity. Whether the effect of grip type on the isolated rotation component is purely biomechanical or also influenced by movement familiarity cannot be answered in the present context.

## 3. Experiment B

### 3.1. Methods

#### 3.1.1. Participants

A sample of 24 male participants with a mean age of 23.4 years (*SD* = 3.3 years) was assessed using the automated apparatus. All 24 participants had either recently received or were currently pursuing a university degree. Handedness was assessed using the Edinburgh Handedness Inventory version by Salmaso and Longoni (1985). One participant was left-hand dominant; all other participants were right-hand dominant. Participants received either study-credits or 20 EUR for their participation. Participants were assigned to one of eight conditions, balancing the order of mixed and blocked sessions, task-cue color assignment (kept constant across sessions for each participant), and the order of blocks in the blocked condition (rule first or plan first). The assignment was matched for age. Instructions were given in German. The experimenter confirmed language fluency.

#### 3.1.2. Materials and procedure

The same equipment and general experimental procedure as in Experiment A was used. The experiments differ in two points. First, the real object version of the RPMC experiment was used in *both* sessions. Second, rather than manipulating action-mode per session (real vs. pantomime) Experiment B sessions varied task switching conditions by presenting either a mixed or a blocked versions of the experiment per session (within-subjects design).

In the blocked version of the experiment, participants received instructions and training trials only for the block that would immediately follow (rule block or plan block) and received the other half of instructions and training trials in the second half of the experiment. In Experiment B half of the participants received the blocked version of the RPMC paradigm in the first session, while the other half received the mixed version first.

#### 3.1.3. Data analysis

The same analyses as in Experiment A were conducted on Experiment B data with the condition factor (mixed/blocked) replacing the action-mode factor (real/pantomime). Thus, the ANOVAs for each of the dependent variables RT, MT, and rotation time, contained the main effects of task (rule/plan), grip (pronated/supinated), hand (non-dominant/dominant), and condition (blocked/mixed) as well as the task^*^condition interaction.

### 3.2. Results experiment B

#### 3.2.1. Reaction time

**Figure 4:**
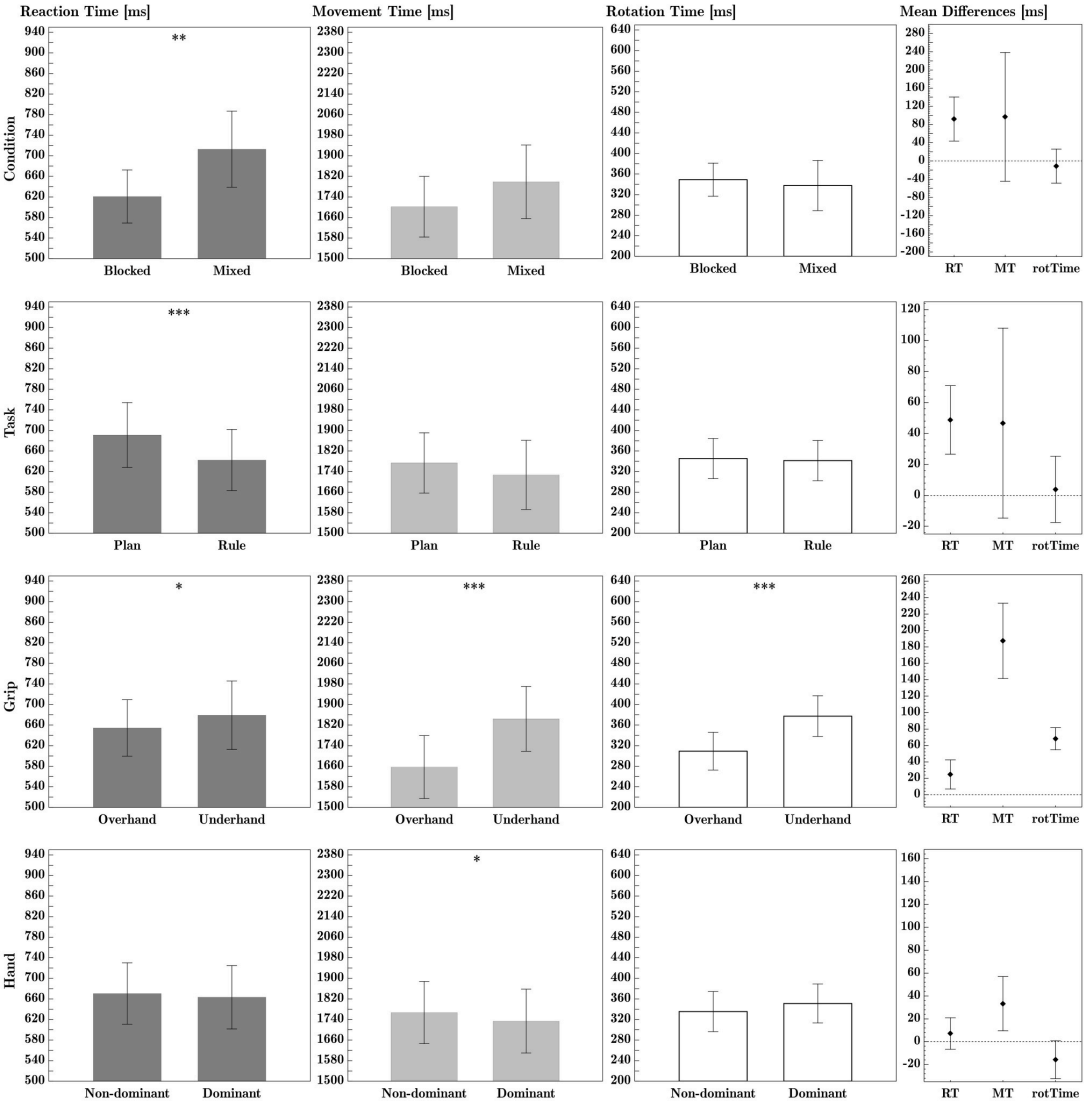
Experiment B ANOVA main effects, for (from left to right column) reaction time, movement time and rotation time. Within-factor differences are shown in the far right column. All times are given in milliseconds. Error bars represent 95% confidence intervals. ^*^, ^**^, and ^***^ denote significant differences with Holm-Bonferroni adjusted *p*-values *p*_*adj*_ < 0.05, < 0.01, and < 0.001, respectively. Calculation of mean difference times: Condition difference = (Mixed − Blocked); Task difference = (Plan − Rule); Grip difference = Underhand − Overhand); Hand difference = Non-dominant − Dominant. See Table 3 for effect sizes and *p*-values.

The ANOVA of RTs showed a significant main effect of task, with participants showing slower mean RTs in the plan task than in the rule task. The planned task comparisons in each condition revealed significant task differences in both the blocked condition, *t*_(23)_ = 2.48, *p* = 0.021, and the mixed condition, *t*_(23)_ = 4.18, *p* < 0.000. There was also a main effect of condition, with slower mean RTs in the mixed version of the experiment than in the blocked version. The significant main effect of grip showed slower RTs for underhand grips than for overhand grips. See Figure 4 for plots of main effects. There was no significant effect of hand on RTs. See Tables 1, 2 for main effect and interaction means, respectively. See Table 3 for comprehensive ANOVA results. Full factorial data are given in Tables S2, S4.

##### 3.2.1.1. Diffusion parameters

Condition had a significant effect on drift rate *v*, with a higher mean rate of information accumulation in the blocked condition than in the mixed condition. The significant effect of task on drift rate, showed higher drift rates in the rule task, than in the plan task. The effect of task on non-decision time with longer *t*_0_ in the mixed condition than in the blocked condition, was not significant after family-wise error correction. Condition^*^task interactions of diffusion model parameters are shown in **Figure 6**. See Table 5 for drift diffusion ANOVA results and Table 4 for drift diffusion parameter means and standard deviations.

#### 3.2.2. Movement time

**Figure 5:**
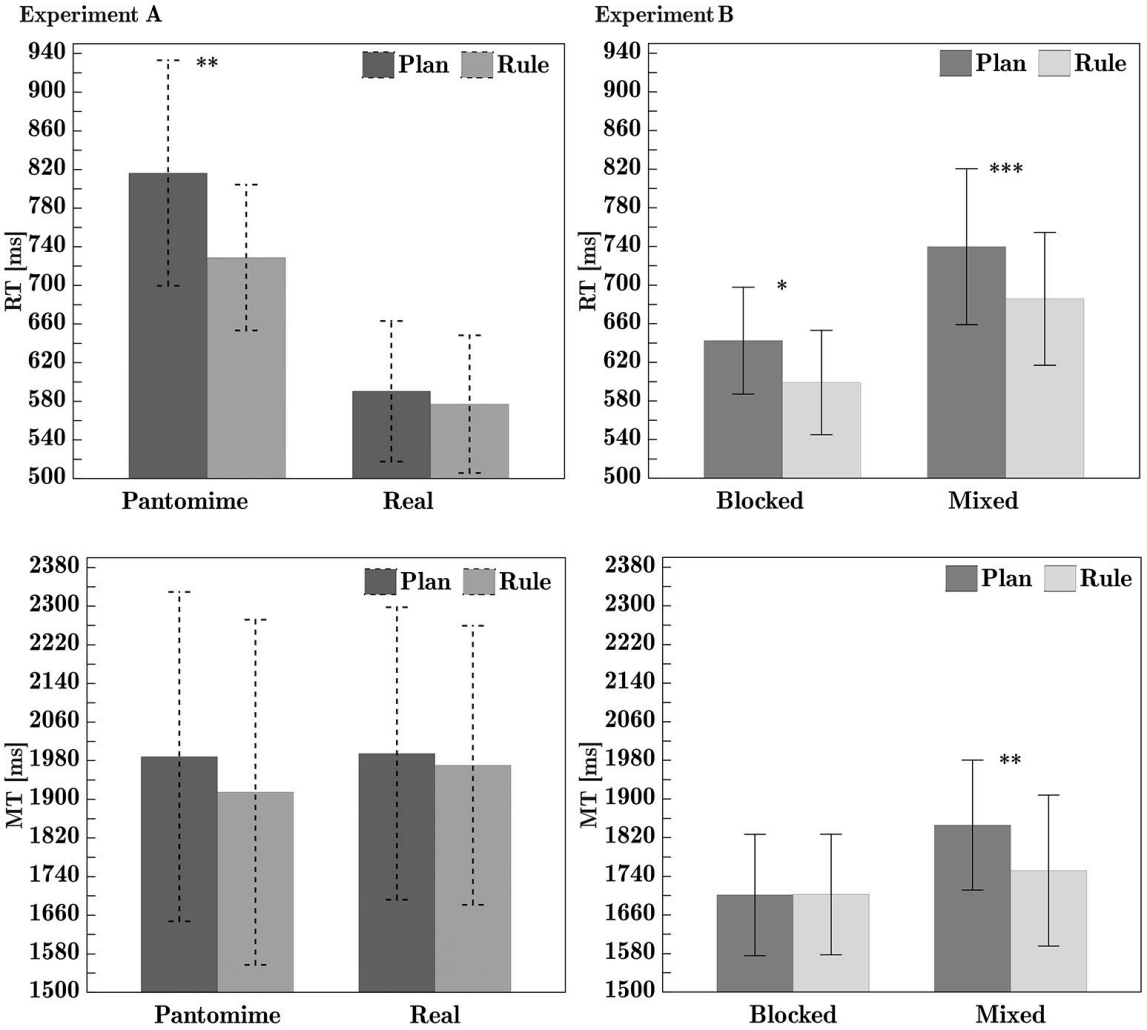
Top row, left panel: Experiment A Mode^*^Task interaction for reaction time. Top row, right panel: Experiment B Condition^*^Task interaction for reaction time. Bottom row, left panel: Experiment A Mode^*^Task interaction for movement time. Bottom row, right panel: Experiment B Condition^*^Task interaction for movement time. All values are given in milliseconds. Error bars represent 95% confidence intervals. Only significant task differences within action-mode and condition are shown. RT differences were tested with t-contrasts. MT differences were tested with Bonferroni *post-hoc* tests. ^*^, ^**^, and ^***^ denote significant differences with *p* < 0.05, < 0.01, and < 0.001, respectively. See Table 3 for interaction effect sizes and *p*-values.

The ANOVA revealed a significant main effect of grip (see Figure 3), with faster MTs in overhand grip trials than in underhand grip trials. Dominant hand MTs were significantly faster than non-dominant hand MTs. See Figure 4 for plots of main effects. The interaction effect between condition and task (see Figure 5), was significant. *Post-hoc* testing indicates that in the blocked condition, there was no significant difference between mean MTs as a function of task, *p* = 1. In the mixed condition however, MTs in the rule task were significantly faster than MTs in the plan task, *p* = 0.005. See Tables 1, 2 for main effect and interaction means, respectively. See Table 3 for comprehensive ANOVA results. Full factorial data are given in Tables S2, S4.

#### 3.2.3. Rotation time

For technical reasons, rotation time data for one session of one participant were not recorded in the data file. That participant was excluded from rotation time analysis. The ANOVA of rotation times showed a significant main effect of grip, with faster mean handle rotation times in overhand trials than in underhand trials. This effect is shown in Figure 3. See Tables 1, 2 for main effect and interaction means, respectively. See Table 3 for comprehensive ANOVA results. Full factorial data are given in Tables S2, S4.

#### 3.2.4. Grip errors

Pair-wise Wilcoxon comparisons showed that the number of grip errors significantly differed only in the plan, 98 total grip errors; *M* = 4.08, *SD* = 3.99, vs. rule, 38 total grip errors; *M* = 1.58, *SD* = 1.79, task comparison, *W*(22) = 22.5, *Z* = 3.38, *p* < 0.001.

### 3.3. Discussion experiment B

In Experiment B we investigated whether task switching affects rule- vs. plan-based efficiency effects in the RPMC paradigm. The $\eta_{p}^{2}$ effect sizes suggest that efficiency effects are dependent on the manipulated aspect that may affect different parts of the movement: movement initiation (RT) seems to be predominantly influenced by task and condition, movement (MT) by condition as well as grip type and hand, and the rotation-movement (rotTime) appears to be predominantly influenced by grip type.

#### 3.3.1. Task and task switching effects

As hypothesized, RTs in the rule task were significantly slower than RTs in the plan task, in line with the results of Randerath et al. (2013, 2015, 2017). Compared to flexible stimulus-response mappings in the plan task, the fixed if-then rules appear to predominantly facilitate the initiation phase of action.

Considering that participants made more errors in the plan task than during the rule task, while simultaneously showing slower reaction times in the plan task than in the rule task, this is strong evidence that the plan task is cognitively more demanding than the rule task. This is further supported by the significantly higher rate of information uptake in the rule task compared to the plan task as quantified by the diffusion parameter *v*.

Effects of task in the later stages of movement (MT) appeared to be modulated by the task switching condition. The significant interaction effect of condition and task on MTs indicates that during high cognitive load (i.e., during the mixed condition), if-then rules appear to reduce MT in comparison to plan-based action-selection. Interestingly, this advantage does not extend to the low cognitive background load condition (i.e., the blocked condition). Haji et al. (2015) used a surgical knot tying task in a study with medical students and measured movement efficiency under different cognitive loads. Students had to solve the task without visual feedback and in a constrained space. Using motion tracking, the researchers found knot tying performance, as measured by the mean number of movements per knot (lower is better) and time per knot (lower is better), to be better in the low cognitive load condition than in the high cognitive load condition. The cognitive load manipulation altered physical parameters of movement. The present experiment extends the evidence for effects of cognitive load on movement parameters by demonstrating the possibility of efficiency effects for relatively simple grasping tasks, i.e., even without the presence of additional constraints or reduced visual feedback.

The hypothesis of faster RTs in the blocked version of the experiment compared to the mixed version of the experiment was corroborated and is best interpreted in the context of task switching. Frequent shifts between cognitive tasks have previously been shown to lead to an increase in RTs and/or error rate (Rogers and Monsell, 1995; Monsell, 2003). The difference between the number of grip errors made in the mixed compared to the blocked version of the experiment was not significant. This could be attributed to the task instructions which placed an increased emphasis on accuracy. As RTs in the mixed condition were longer than in the blocked condition it appears that the speed-accuracy trade-off (e.g., Fitts, 1966; Ollman, 1966; Pachella and Pew, 1968) was skewed toward accuracy. Contrary to expectations our diffusion model data failed to significantly account for task switching costs in the *t*_0_ parameter after family-wise error correction. However, we did find the predicted higher drift rate in the blocked condition compared to the mixed condition. The effect of task switching on drift rate has previously been discussed by Schmitz and Voss (2012, 2014).

#### 3.3.2. Effects of hand and grip type

As in Experiment A, there were main effects of hand and grip on MTs with faster dominant than non-dominant hand MTs and slower underhand than overhand MTs. Also as in Experiment A, rotation times of underhand grips were significantly slower compared to overhand grips. However, counter to Experiment A and in line with Randerath et al. (2013), we found the predicted RT difference between overhand and underhand grips.

#### 3.3.3. Experiment B conclusion

**Figure 6:**
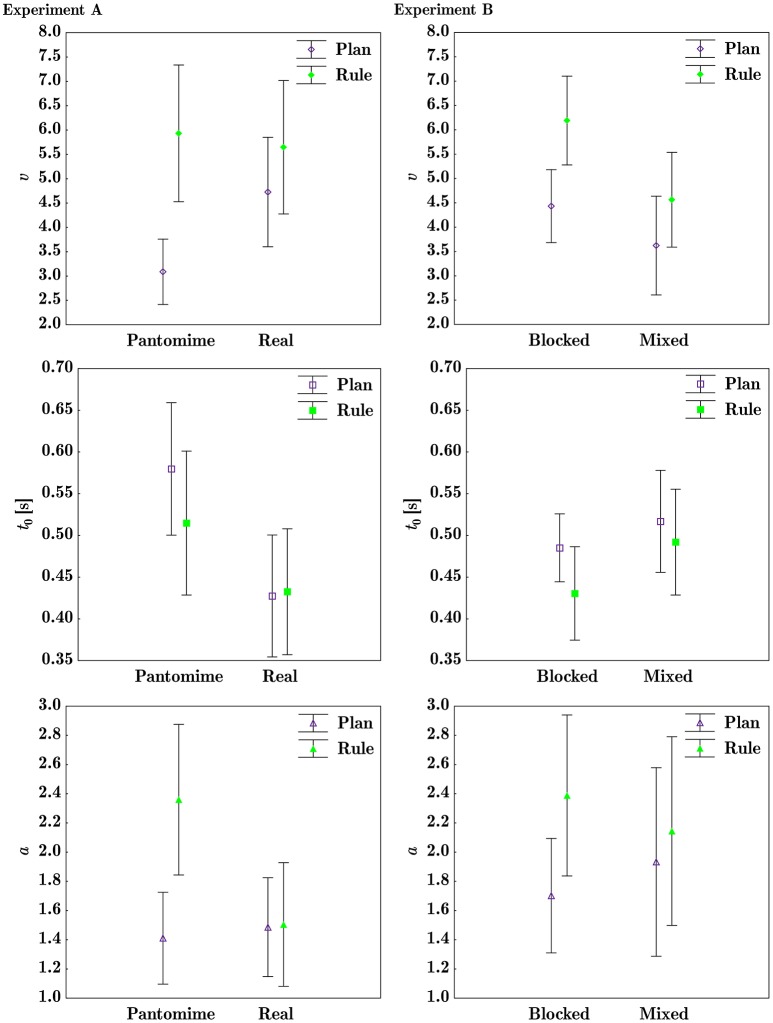
The left column shows Experiment A action-mode^*^task interactions; the right column shows Experiment B condition^*^task interactions for drift rate *v* (first row), non-decision time *t*_0_ (in seconds, second row), and decision boundary separation *a* (bottom row). See Table 5 for effect sizes and *p*-values.

Compared to Experiment A, in Experiment B the evidence for task based efficiency effects when interacting with real objects appeared stronger. While drift rates showed a similar pattern in both experiments, we found significant efficiency effects of rule-based action planning captured by reaction times in real object interactions, in Experiment B but not A. One key difference that could explain the larger task difference in RTs in Experiment B, compared to the Experiment A real condition, may lie in the way participants accomplished the tasks. In the following we provide a potential explanation. Firstly, the findings indicate that a portion of the grip selection process in the Experiment A real action-mode may have carried over into the movement phase, and thus reduced the effect of task in real action-mode RTs. An argument for less discrete grip selection during action initiation in Experiment A, is supported by the missing RT difference between overhand and underhand grips in Experiment A that subsequently appears to be present in the MT parameter. In line with this, Figure 5 displays shortened RTs and prolonged MTs in the Experiment A real action-mode compared to the Experiment B mixed condition. Secondly, response caution (*a* parameter) in the Experiment A real action-mode compared to the Experiment B mixed condition appears to be lower (see Figure 6). This may indicate that participants in Experiment B responded more cautiously, going along with a larger potential for efficiency effects to show. This suggests that Experiment A participants, on average, placed more emphasis on the speed aspect of the task instructions than Experiment B participants. In line with this argument, it has been shown that the reduction in boundary separation is sensitive to the emphasis on speed (e.g., Zhang and Rowe, 2014). Future research might make use of response caution manipulations to determine the bounds of rule-based efficiency effects as they relate to individual response criteria.

## 4. General discussion

The RPMC paradigm demonstrates that for visually indistinguishable grasp-to-rotate actions, implementation intention based rules can induce quicker processing compared to the instruction to achieve a comfortable end position. However, although, measures of efficiency appear to produce a consistent pattern of results (i.e., shorter rule than plan RTs, higher rule than plan drift rates) the magnitude of this efficiency effect does not appear to be entirely robust across action-modes, task-switching conditions or measured parameters. Instead, the magnitude of the facilitating effect seems to be highly dependent on the present cognitive load.

Decomposition of RTs using diffusion modeling showed that the added imagery component in the pantomime task is well captured in the relative increase of the non-decision time parameter while accounting for changes in both rates of information accumulation and response criteria. Furthermore, diffusion modeling allowed us to describe how efficiency of the grip selection process is affected by the task manipulation, while controlling for other processes contained in reaction times, as well as individual response criteria.

As expected, we observed advantages of rule-based over plan-based responses in both the pantomime and real object condition (Experiment A) as well as within a real object mode for both a blocked and a task switching condition (Experiment B). However, this effect appeared stronger under conditions for which enhanced difficulty is suggested: the pantomime action-mode and the task-switching mode, respectively. Further, in Experiment A, higher efficiency of rule-based action was only found for RTs in the pantomime action-mode, but not in the real object action-mode. However, drift rates appeared to be more sensitive in detecting differences between tasks across action-modes. As expected, participants showed significantly higher drift rates *v* in the rule task than in the plan task, supporting the notion of facilitated processing in the fixed response mapping condition irrespective of action-mode and task-switching condition.

Given the described variability of efficiency effects modulated by cognitive load, the question arises what this means with respect to the idea of a meaningful application of implementation intention based rules to facilitate motor cognitive tasks in the rehabilitative context of stroke patients. Determining components that can be manipulated in stroke patients to facilitate active behavior is critical to neurorehabilitation and may foster use-dependent plasticity (Kimberley et al., 2008). Stroke can go along with impaired cognitive functions and marked slowness of information processing (Hochstenbach et al., 1998; De Luca et al., 2017). It can compromise motor cognitive tasks affecting activities of daily living (Goldenberg, 2013; Buchmann and Randerath, 2017). We argue that implementation intention based rules may be particularly effective in improving the successful selection of actions in patients with difficulties therein. Thus, even though in healthy young adults efficiency effects of implementation intention based rules in the RPMC paradigm are reduced in actions involving real objects, patients with difficulties in action planning may still profit significantly. We propose that by implementing such rules the load on the already limited cognitive resources may be reduced in patients with stroke. Spared cognitive resources may thereby be used to support active behavior. Whether these efficiency effects simply reflect a reduction of load on the same processes or whether both approaches to action target different mechanisms needs further clarification. In any case, both mechanisms could be helpful. Future studies including clinical populations will have to investigate this point.

The utility of rule-based approaches seems likely, for example, for limb apraxia patients with parietal lobe lesions and associated deficiencies in planning based movements. The idea is underpinned by a recent functional imaging study that demonstrated that rule-based actions put less strain on neural networks of action- selection particularly in parietal regions (Randerath et al., 2017).

Intriguingly, studies which have combined imaging methods with diffusion modeling have found evidence accumulation correlates in parietal regions (e.g., van Vugt et al., 2012), and frontoparietal networks (for a review see Mulder et al., 2014). Future imaging studies using the RPMC paradigm could use diffusion model-based decomposition of task performance to localize process specific neural substrates, as has been done in other high-level cognitive neuroscientific subfields (for an overview see Forstmann et al., 2016). Doing so may aid in the identification of patient populations that could benefit from rule-based action planning.

Lastly, the present research contributes to the literature on implementation intentions by investigating motor planning in a controlled laboratory setting, and thereby fills a crucial gap in the literature. On the one hand, past laboratory research on implementation intentions has largely relied on intellectual or cognitive tasks such as reacting to stimuli with button presses or evaluating pictures and recent studies using physical tasks (Bieleke and Wolff, 2017; Thürmer et al., 2017) focused on endurance performance only. On the other hand, applied implementation intention research has largely investigated complex behaviors, such as eating more healthily (Adriaanse et al., 2011; Vilà et al., 2017). The present research investigates basic motor planning in the laboratory with a task that closely resembles a real challenge for neuro-rehabilitation patients: Grasping and interacting with an object. Thereby, this research combines high external validity with high experimental control. Moreover, the present research contributes to the recent efforts of modeling how exactly implementation intentions achieve performance improvements (Stewart and Payne, 2008; Janczyk et al., 2015).

## Author contributions

JS: Analysis, interpretation, study design, apparatus development, data acquisition, and writing. SS: Data acquisition, study design, analysis, writing. JT and JR: Study design, interpretation, writing.

### Conflict of interest statement

The authors declare that the research was conducted in the absence of any commercial or financial relationships that could be construed as a potential conflict of interest.
