# Supplementary material for: Efficiency in Rule- vs. Plan-Based Movements Is Modulated by Action-Mode
Source: Front Psychol. 2018 Mar 13;9:309. doi: 10.3389/fpsyg.2018.00309 (PMC5859074; doi:10.3389/fpsyg.2018.00309)
Supplement: Supplementary file 1 [file Image1.PDF]

Supplementary Material: Efficiency in Rule- vs. Plan-Based Movements is Modulated by Action-Mode

Jean Patrick Philippe Scheib *, Sarah Stoll, J. Lukas Thürmer, and Jennifer Randerath

*Correspondence:
Jean Patrick Philippe Scheib
j.p.p.scheib@gmail.com

In the following, details pertaining to the rotation apparatus used in Experiment B will be given. Updated schematics and source code will be made available on http://github.com/MoCogKonstanz/RPMC.

1 RPMC APPARATUS

An automated apparatus was built with the aim of increasing experimental efficiency by increasing both the frequency of trial presentation by eliminating the need to manually set the handle, and by increasing the accuracy of pre- and post-rotation handle angles, thereby improving between-trial consistency. Furthermore, the apparatus enables the measurement of the duration of participants' rotational handle manipulations, which have previously not been measured in the RPMC context.

1.1 Functional principle

A list of 128 numbers (one for each trial) ranging from 1 to 16, coding for the 16 different trial types, was programmed into SuperLab's digital output function.

Prior to each trial SuperLab sends the trial information to the "timing Teensy" via a National Instruments NI-6501 USB digital acquisition device (DAQ)[1], coded to five digital I/O lines using high/low logic, with logic changes triggered by falling edge (changes from high to low voltage) combinations of the lines (essentially, binary number coding for numbers 1 to 16. The timing Teensy then relays the information to the "control Teensy" via serial communication at a baud rate of 115200 Bd.

The control Teensy in turn, forwards the information to the handle positioning subroutine which positions the handle and then turns off the stepper motor, enabling resistance free turning of the handle. A switch case statement utilizes the trial information to set the appropriate color combination of the through-hole LEDs positioned near the left and right edges of the handle.

As soon as the handle's deviation from the predefined start angle (45° or 315°) is greater than the preset tolerance of 2.8125°, the control Teensy sets the timing start trigger pin to low (from 5V to 0V). This voltage change is read by the timing Teensy. The timer on the timing Teens begins incrementing with submillisecond resolution until the control Teensy sends a stop trigger by setting the timing stop trigger pin to low, when the target angle is reached. The stop trigger also induces a reading of the capacitive touch

[1] The DAQ was also used to control the PLATO goggles on the same I/O port on different channels. Running the apparatus and the goggles on the same port was necessary as SuperLab appeared to have problems maintaining port integrity (i.e., signals sent to control the goggles also influenced the apparatus).

sensors wired to the timing Teensy, which are used to differentiate between overhand and underhand handle grasps. As soon as the stop trigger is sent, the control Teensy reactivates the stepper motor and sends an ”active stop” command to the motor preventing further rotation of the handle and turns off the LEDs, giving participants tactile and visual feedback that the target has been reached. After completion of the trial (i.e., participants return the active hand to the response pad), SuperLab requests rotation time and touch sensor information from the timing Teensy, using a unique falling edge combination of digital I/O lines. The timing Teensy, by emulating a USB keyboard, then sends an ASCII string back to SuperLab and waits for new trial information from SuperLab.

1.2 Development

The RPMC apparatus was designed with as many off-the-shelf components as possible in order to expedite development and facilitate replicability by other researchers (visit http://github.com/MoCogKonstanz/RPMC for wiring schematics). The apparatus can be viewed as a two-part device consisting of a control unit which houses most of the electronic components and an input unit with which participants actually interacted.

The RPMC apparatus is built around two PJRC Teensy 3.1 microcontrollers (http://www.pjrc.com) overclocked to 96 MHz, running firmware programmed in Arduino (http://arduino.cc) available under (https://github.com/MoCogKonstanz/RPMC). The use of previous versions of the PJRC Teensy to record participant responses in psychological experiments has been described by Rorden and Hanayik (2014) and evaluated favorably in terms of input latency (M = 2ms, SD = 0.54 ms) and comparability to dedicated professional input device solutions.

Other Arduino compatible microcontrollers have been used in a psychological context to build MRI compatible computer-controlled systems for olfactory stimulation (Andrieu, Bonnans, Meneses, Millot, Moulin, & Gharbi, 2014), fMRI-Compatible input devices (Hollinger, Steele, Penhune, Zatorre, & Wanderley, 2007; Rorden & Hanayik, 2014), automated Operant conditioning chambers (Pineño, 2014; Escobar & Pérez-Herrera, 2015) and advanced response boxes capable of measuring hardware-side response latencies (Schubert, D'Ausilio, & Canto, 2013).

More generally, accuracy tests conducted by D'Ausilio (2012) show that Arduino boards are a viable and inexpensive tool for psychological and neurophysiological research.

1.2.0.1 Control unit

Besides the two microcontrollers, the aluminum 249 x 66 x 48 mm (width x depth x height) control unit (see Fig. S1 A to F) also houses a dedicated stepper motor controller (EasyDriver v4.5; http://www.schmalzhaus.com/EasyDriver/), powered by an external 5V power supply, which allows for precise motor control using the AccelStepper library (http://www.airspayce.com/mikem/arduino/AccelStepper/). Components were hand soldered onto a breadboard and electrically insulated from the aluminum housing by a sheet of cardboard. The firmware makes use of existing libraries for stepper motor control (AccelStepper), software debouncing of input buttons (Bounce), capacitive sensing (CapacitiveSensor; used for grip detection) and pulse-width modulated LED control (Adafruit_NeoPixel; used to control the integrated circuit of the NeoPixel LEDs), thus further reducing development time.

As a semi-automated version of the apparatus using the same hardware was developed, input buttons and radial selector switches were added to the input unit housing. The semi-automated version of the device automatically set handle start positions and light combinations according to a predefined list embedded in a

version of the firmware specific to that configuration and required manual block and experiment version selection as well as trial selection (using a ”next” and ”previous” trial button). Considerations pertaining to clock issues during hardware interrupts[2] triggered by input buttons as well as memory issues caused by integer arrays, which contained the trial lists in the semi-automated configuration, were the primary reasons for the dual microcontroller configuration which was finally implemented.

1.2.0.2 Input unit

Essentially, the input unit (see Fig. S1 H to J) consists of a 381 mm (15 in.) Actobotics (http://www.servocity.com/html/actobotics.html) aluminum channel containing a NEMA 11 stepper motor and a 5:1 geared transmission which rotates the 3D-printed cylindrical handle once for every 1800° of motor rotation and the precision wirewound 10KΩ, 3600° linear potentiometer, which was used to measure the handle’s angular position, once for every full rotation. The input unit was screwed onto a 200 x 400 x 33 mm (width x depth x height) wooden base to prevent toppling and slipping of the unit. The aluminum channel and the wooden base were wrapped in matte grey self-adhesive foil to create a non-distracting uniform appearance.

Capacitive touch sensor arrays on top and bottom of the handle were made using strips of self-adhesive copper tape soldered together to facilitate conductivity within the sensor array and covered with black electrically insulating tape to minimize the possibility of static discharge upon skin contact. This has the added benefits of obscuring the sensors from view and preventing oxidation caused by contact with sweat, which could have asymmetrically altered touch induced capacitance readings and thus been detrimental to grip sensing. The touch sensors and LEDs were connected to the control Teensy via a 12 wire cylindrical slip ring contact embedded in the handle’s shaft, which allowed full and unimpeded rotation of the handle. The LEDs used (pulse-width modulated Adafruit NeoPixel diffused glow 8 mm through-hole RGB LEDs with a WS2811 integrated circuit on-board) were chosen for their clear visibility and diffuse glow properties.

Early prototypes of the handle were made from pieces of 200 mm long polyvinyl chloride (PVC) tubes of 50 mm diameter. Drilling properly aligned holes for the LEDs and the 152.4 mm (6 in.) aluminum hollow shafting (aluminum tube of 12.7 mm or 0.5 in. diameter) terminal, using hand-held power tools proved too inaccurate to be considered a practicable solution. With the assistance of a colleague fluent in Computer Aided Design (CAD) a 3D model of the handle was created and 3D printed by a third-party 3D printing service out of acrylonitrile butadiene styrene (ABS) with holes for the through-hole LEDs and shafting terminal already in place and properly aligned, requiring no additional modification. The handle is the only part of the apparatus not readily available ”off-the-shelf”.

Though not objectively measured, torque required for handle rotation is minimal. This was achieved by mounting all shafting in pillow blocks with embedded bearings of appropriate diameter.

1.3 Validation

As the RPMC apparatus was first utilized in the present experiment, it is necessary to analyze several properties of the apparatus pertaining to the reliability of measurements. This is necessary in order to discover potential design flaws and generally improve upon all relevant properties of the apparatus in future iterations. One of those properties, namely input latency or rather the variability thereof, in input devices used in psychometric experiments, is of increasing concern in the psychological community, as they have been shown to influence results in an unpredictable manner (Ulrich & Giray, 1989; Plant & Turner, 2009). Generally, measurement of a very large number of trials is required to get a proper approximation of true

[2] The microcontroller clock does not increment during hardware interrupts.

precision and accuracy. Due to the relatively large file size of high-speed video and the resource consuming nature of video analysis, 128 validation trials wer performed. Using the terminology proposed by Plant and Turner (2009) the term *accuracy* will be used to refer to the degree to which measured rotation times correspond to actual rotation times and *precision* to describe the dispersion of measurements around the mean (i.e., high precision equals low standard deviation).

1.3.0.1 Timing accuracy and precision

To assess timing accuracy and precision of rotation times, 6 trials were recorded using a CASIO EX-F1 high speed camera recording video at 1200 frames per second (fps). The number of frames from beginning to end of the rotation was summed up and converted to milliseconds using the following formula:

$$T_{\text{rotTime}} = \frac{\sum_{\mathbf{x_i}=m}^{n} \mathbf{x_i}}{(F_{\text{rate}}/1000)}$$

where T_{rotTime} is the rotation time in milliseconds; $\mathbf{x}$ a row vector containing all frames capturing the rotation, from the first frame m to the last frame n, as components i each with a value of 1; and F_{rate} is the camera's frame rate of 1200 fps.

The calculated values of T_{rotTime} were then compared to the corresponding rotation times reported by the apparatus to estimate measurement error.

1.3.0.2 Grip detection accuracy

Participants' use of pronated and suppinated handle grasps were recorded on video and manually in SuperLab by the experimenter after every trial. Those records were compared to the corresponding sensor data reported by the apparatus. If automated grip detection should prove to be sufficiently accurate, manual recording of participant responses could be dispensed with, which would allow the possibility of largely unsupervised testing, thus minimizing the impact of possible experimenter effects and reducing experimenter demand.

2 VALIDATION RESULTS

2.1 Timing accuracy and precision

Using frame-by-frame analysis of high-speed video data, a mean discrepancy of 28.37 ms (*SD* = 26.09 ms) was calculated between rotation times measured by the apparatus and rotation times calculated from video data. Rotation times reported by the apparatus were shorter than calculated rotation times.

2.2 Grip detection accuracy

For one session of one participant, no rotation time or grip detection data were recorded in the data file.

Including those 128 trials, with missing data counted as erroneous grip detection, automated grip detection achieved an accuracy of 92%. It is however unclear which link in the data acquisition chain is responsible for the missing data. As such, the 92% accuracy rating should be considered the lower bound of achievable accuracy. The upper bound of grip detection accuracy was calculated by excluding the 128 trials with missing data from the total number of trials, making a total of 6016 trials. As 5636 grasps were correctly classified, this results in an upper bound accuracy calculation of 94%.

Real-time grip coding from visual observation was however 100% accurate as confirmed by comparison with video data.

3 APPARATUS DISCUSSION

3.1 Grip detection

94% of grasps were correctly identified by the apparatus. This means that on average, the apparatus classified almost eight out of 128 grasps per individual session falsely. Despite this high error rate, the present implementation of automated grip detection may be useful under certain experimental conditions. For example, when experimenter effects may be of particular concern. It remains to be seen in how far improvements in firmware and sensor array layout can improve automated grip detection using capacitive sensing, within reasonable cost.

3.2 Reliability and improvements

In 6144 trials the apparatus produced 28 errors involving the LEDs and stopped significantly short of the target angle 186 times. Which means that the apparatus did not perform correctly in 3% of trials. Moreover, analysis with high-speed video revealed that there is much room for improvement in terms of timing accuracy and precision.

The LED light errors were nearly exclusive to the left LED, which received its pulse-width modulated signal directly from the integrated circuit of the right LED. Possibly, due to interference within the slip ring contact or from hardware interrupts on the microcontroller, or wire length between LEDs, the right LED sent erroneous lighting color information to the left LED. As an additional slip ring line is free, the left LED can easily be controlled directly from the micro controller, which should eliminate the problem.

Errors in which the apparatus stopped rotations significantly short of the target do not only reflect inaccurate rotation time measurements but also affect movement time (movement times become shorter when the angle of rotation is less) and must thus be completely eliminated. Those errors were most likely caused by wear on the potentiometer used to measure handle angles, as those errors first appeared after the eleventh data set was collected and steadily increased from then on. The potentiometer consists of a resistance element and a wiper. Each rotation slowly wears down those two components. Due to the 5:1 transmission, the potentiomter is rotated 450° in each trial at five times the speed of the handle rotation. The mean angular velocity of the handle was 43.54 rpm and 217.68 rpm for the potentiometer shaft. Though maximum angular velocity was certainly higher than mean angular velocity, it is interesting to compare orders of magnitude for reference: An LP record rotates at a speed of $33^1/_3$ rpm, diesel engines in ships run between 60 and 200 rpm and helicopter blades during flight spin at approximately 190 to 400 rpm. Counter-clockwise rotations increase ohmic resistance of the potentiometer. Resistance is increased by a thickening of the resistance element. As such, one would predict more wear in exactly those areas. Increased wear and the accumulation of metallic debris from that wear seem to have increased the likelihood of contact loss between wiper and resistance element, and inaccurate resistance measurements, respectively.

To prolong the working-life of the wire potentiometer, a 1:1 gearing could be implemented between the handle and the potentiometer (and even the motor). Doing so would mean five times as few rotations at a fifth of the angular velocity, which would certainly have positive consequences for potentiometer life-span. Furthermore, the potentiometer could then be rotated, so as to shift worn areas of the resistance element multiple times, as each rotation would only cover $^1/_{40}th$ of the maximum potentiometer rotation angle (3600°) instead of $^1/_8th$, requiring only re-calibration instead of a replacement potentiometer. Also, the

reduced potentiometer rotation angle would allow for the use a cheaper and more rugged non-multi-turn potentiometer.

Analysis using high-speed video recording indicates rather poor accuracy and precision of handle rotation time measurement. Fortunately the large number of trials in the present experiment likely allowed measurement error to average out. The discrepancy between actual rotation times and measured rotation times was caused by the length of the main loops in the Teensy firmware and delays caused by updating pin states. That problem has been solved by moving SuperLab-RPMC to the control Teensy and using the timing Teensy main loop only to measure the interval between start and stop triggers.

Possible firmware improvements aside (i.e., reducing the size of the main loop by outsourcing functions to subroutines, making use of hardware interrupts, and mapping the AnalogRead function to a higher resolution), other simple hardware-side improvements include the application of epoxy-resin-based glue, to areas where grub screw clamps are used, to minimize clamp slippage and thereby increase the interval between calibrations.

Further, capacitive grip sensing could be improved by optimizing sensor layout and introducing capacitors for de-noising. The apparatus greatly minimizes experimenter work-load and allows the experimenter to focus all attention on the observation of participant movements, which facilitates accurate grip coding by the experimenter. The utility of automated grip detection thus seems limited to situations where multiple participants are assessed at the same time and sufficient numbers of trained experimenters are not available.

3.3 Conclusions and Limitations

Given the relatively short development time and limited pre-testing, the apparatus generally performed well. The main objectives of improving trial-by-trial consistency and limiting experiment duration by automatically setting handle angles were achieved. Automated grip detection and rotation time measurement on the other hand are in need of improvement. The relatively low precision of handle rotation time in particular, requires significant improvement to allow unbiased measurement of motor-cognitive ability. Approaches for the rectification of present apparatus short-comings were given in the previous section.

4 IMPROVEMENTS

See http://github.com/MoCogKonstanz/RPMC for schematics and firmware of the improved apparatus used in Experiment A. Rotation-time measurement was considerably improved (rotation time discrepancy: M = 3.1 ms, SD = 1.8 ms). This was primarily accomplished by implementing hardware interrupt routines, and transferring light control and interfacing to one microcontroller, allowing the other controller to more accurately measure time. As it does not seem particularly useful for our current research endeavours, we have discontinued automated grip detection, and it is no longer implemented in the present version of the apparatus.

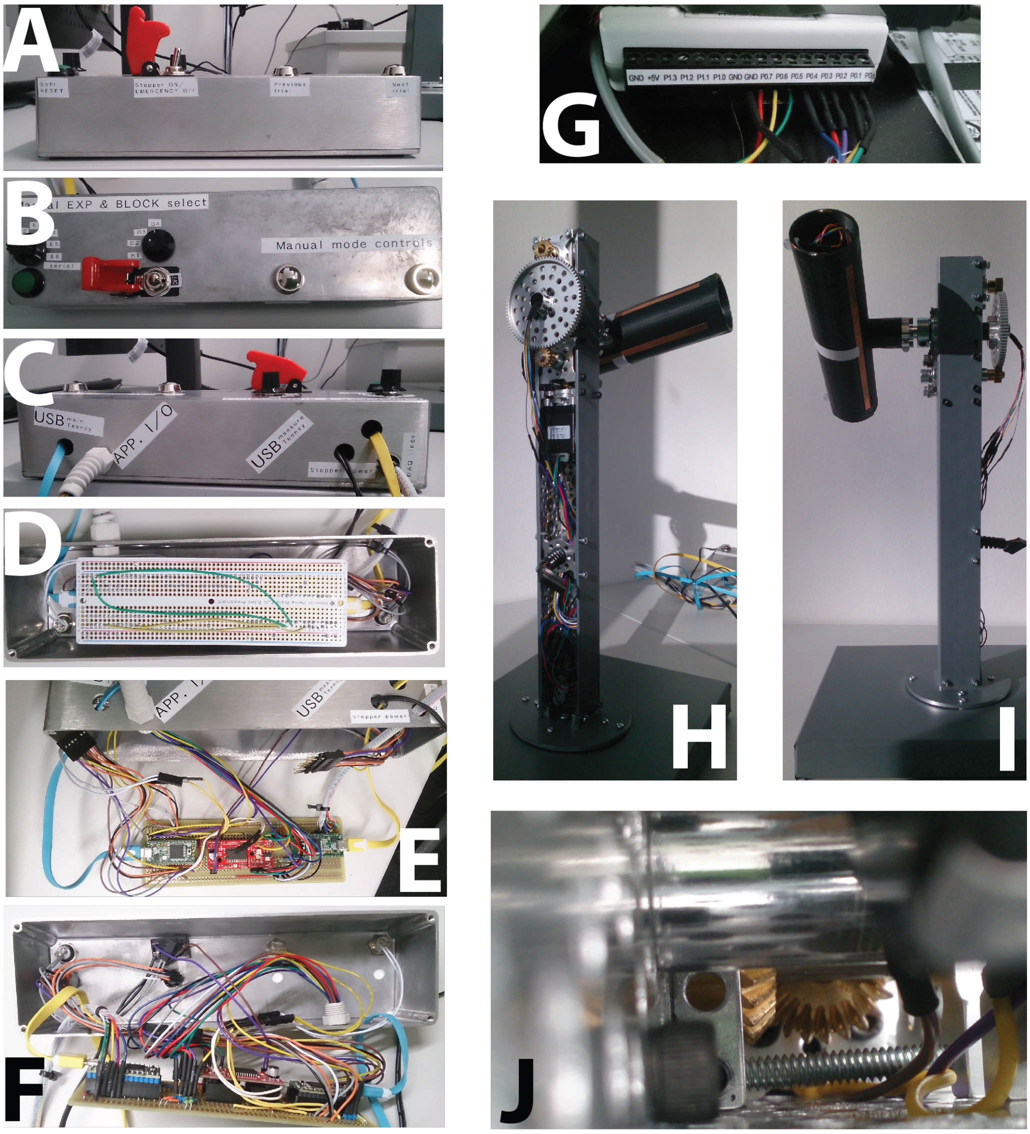

Figure S1. (**A**) a frontal view of the control unit with manual mode controls. First row from left to right: reset button, stepper motor power switch, previous trial button, and next trial button. Second row from left to right: rotary switch for experiment version selection, rotary switch for block selection.; (**B**) shows a top view of the control unit (see **A** for button and switch descriptions); (**C**) shows a rear view of the input unit. Cables from left to right: Control Teensy USB cable, apparatus/control unit interface cable, stepper controller power supply cable, timing Teensy USB cable, and DAQ interface lines. (**D**) shows a bottom view of the control unit (without the floor plate) and the underside of the breadboard.; (**E**) shows a top view of the breadboard and disconnected connectors for next trial button (white) and rotary switches. Microcontrollers on the breadboard from left to right: Control Teensy, stepper motor controller, and timing Teensy.; (**F**) shows a side view of the breadboard with microcontrollers mounted on female headers to allow for easy replacement.; (**G**) shows digital acquisition device (DAQ) ports and DAQ interface wires bundled in woven polyethylene terephthalate (PET) sleeve (right wire bundle), and visual occlusion goggle wires (left wire bundle).; (**H**) shows a rear view of the input unit. Gears from top to bottom: 16 tooth copper pinion gear attached to potentiometer shaft, 80 tooth aluminum gear attached to handle's aluminum hollow shaft, and 16 tooth copper pinion gear attached to 6.3 mm ($\frac{1}{4}in.$) motor transmission shaft.; (**I**) shows a side view of the input unit with slip ring contact wires exiting the handle shaft, and exposed rear sensors of top and bottom sensor arrays, used for capacitive grip sensing. (**J**) shows an inside view of the main aluminum channel photographed from the top. Note the routing of potentiometer wires away from the aluminum hollow shaft and bevel gear transmission.

Table S1 Diffusion Model Simulation Results

Experiment	Data set	-LL fit index	Percentile rank	5%-quantile	95%-quantile
Exp. A	1	-163.19	53.80	-279.10	-35.93
	2	-147.95	58.00	-261.39	-39.37
	3	-111.56	43.30	-201.10	17.09
	4	-135.91	60.60	-241.80	-42.71
	5	-7.19	53.80	-140.81	146.51
	6	-279.64	49.50	-365.26	-187.26
	7	-238.60	67.00	-336.42	-175.67
	8	-270.56	48.90	-360.46	-172.98
	9	-198.42	70.70	-346.12	-114.48
	10	-192.47	49.40	-279.34	-85.22
	11	-187.06	47.80	-272.78	-70.42
	12	-179.33	54.10	-282.02	-58.07
	13	-279.64	46.70	-368.72	-173.91
	14	-177.95	45.30	-271.74	-38.42
	15	-149.31	52.80	-269.75	-6.49
	16	-300.17	54.10	-382.09	-225.46
Exp. B	1	-81.10	55.60	-188.46	26.85
	2	-231.74	52.00	-341.39	-127.63
	3	-126.74	52.70	-247.35	7.23
	4	-281.71	41.70	-351.44	-187.01
	5	-82.34	55.40	-204.92	55.59
	6	-300.98	28.80	-361.05	-158.63
	7	-193.04	46.60	-298.81	-60.38
	8	-139.50	56.30	-261.10	-19.66
	9	-179.11	45.70	-269.15	-47.06
	10	-260.49	42.10	-335.39	-157.84
	11	-231.10	47.90	-316.13	-117.77
	12	-234.83	49.40	-333.14	-127.35
	13	16.94	47.40	-76.42	170.29
	14	-86.68	55.70	-205.86	40.28
	15	-290.48	39.00	-372.92	-176.71
	16	-218.43	47.30	-313.43	-97.96
	17	-217.79	35.50	-287.47	-84.58
	18	-297.85	34.80	-368.98	-171.29
	19	-40.50	67.00	-203.38	79.34
	20	-116.56	66.10	-246.16	-22.75
	21	-220.94	49.80	-320.40	-108.07
	22	-280.90	34.20	-351.78	-153.87
	23	-245.44	49.90	-335.17	-131.00
	24	-284.90	38.90	-362.45	-168.46

Note. The table gives percentile ranks of Experiment A and B model fits per participant. Quantiles were calculated from 5000 simulated data sets, calculated from empirically derived parameter sets (see Diffusion model analysis and simulation study paragraph in the main text for details). All empirical parameter fits were penalty-free. Model fits and quantile cut-offs are given as negative log likelihood values (lower indicates better fit).

Table S2 Full Factorial Means and Standard Deviations

Experiment	Mode	Task	Grip	Hand	RT		MT		rotTime	
					M	*SD*	*M*	*SD*	*M*	*SD*
Exp. A	Pantomime	Plan	OH	ND	796.1	203.4	1937.3	597.3		
				Dom	813.8	217.8	1877.3	609.5		
			UH	ND	859.4	240.7	2105.7	618.4		
				Dom	796.0	225.1	2032.8	699.8		
		Rule	OH	ND	763.0	156.8	1924.4	636.5		
				Dom	694.5	106.4	1853.5	581.5		
			UH	ND	730.4	153.6	1982.2	692.0		
				Dom	727.3	166.2	1898.9	711.7		
	Real	Plan	OH	ND	576.0	119.9	1995.0	593.2	405.3	193.4
				Dom	584.9	131.1	1843.2	474.8	375.9	163.5
			UH	ND	594.8	153.4	2140.0	616.1	489.1	186.2
				Dom	606.0	162.8	2001.2	565.0	474.0	207.1
		Rule	OH	ND	576.0	140.8	1971.6	528.7	398.0	188.1
				Dom	575.7	140.0	1806.8	473.1	382.2	170.9
			UH	ND	581.7	158.8	2078.9	614.0	473.6	192.8
				Dom	575.1	133.4	2024.4	569.5	453.8	172.3
Exp. B	Blocked	Plan	OH	ND	625.9	116.9	1664.6	308.5	313.4	105.9
				Dom	632.3	127.6	1574.8	314.1	298.1	86.7
			UH	ND	664.3	152.2	1779.8	338.5	357.6	107.8
				Dom	647.5	153.4	1786.1	308.7	412.2	147.9
		Rule	OH	ND	600.0	121.0	1656.7	307.1	319.1	89.3
				Dom	583.5	117.7	1570.6	297.3	306.4	98.4
			UH	ND	608.7	146.0	1798.6	309.6	354.8	92.6
				Dom	604.3	138.7	1784.2	316.8	433.8	154.8
	Mixed	Plan	OH	ND	714.4	170.1	1750.0	323.9	328.0	103.5
				Dom	728.1	177.6	1720.4	342.9	314.3	116.9
			UH	ND	762.5	215.7	1956.7	414.6	362.2	129.3
				Dom	754.4	217.2	1956.9	401.8	378.2	131.6
		Rule	OH	ND	687.5	157.8	1689.9	403.8	311.5	114.9
				Dom	664.0	167.8	1626.4	406.2	295.1	117.4
			UH	ND	699.8	179.8	1839.8	401.8	353.3	117.5
				Dom	691.6	186.8	1850.3	420.3	374.7	145.9

Note. The table shows means (*M*) and standard deviations (*SD*) of Experiment A and B reaction time (RT), movement time (MT), and rotation time (rotTime) data in milliseconds. ND = non-dominant hand, Dom = dominant hand, OH = overhand grasp, UH = underhand grasp.

Table S3. Experiment A Full Factorial ANOVA Results

Effect	RT			MT			rotTime		
	$F(1, 15)$	p	η_p^2	$F(1, 15)$	p	η_p^2	$F(1, 15)$	p	η_p^2
Intercept	375.27 ***	.000	0.96	190.47 ***	.000	0.93	98.05 ***	.000	0.87
Mode	33.99 ***	.000	0.69	0.17	.684	0.01			
Task	9.51 **	.008	0.39	16.60 ***	.001	0.53	1.39	.257	0.08
Grip	2.09	.169	0.12	18.57 ***	.001	0.55	27.41 **	.000	0.65
Hand	2.35	.146	0.14	16.05 **	.001	0.52	1.77	.203	0.11
Mode*Task	10.12 **	.006	0.40	1.80	.200	0.11			
Mode*Grip	0.00	.986	0.00	2.14	.165	0.12			
Task*Grip	1.67	.216	0.10	3.99	.064	0.21	0.91	.354	0.06
Mode*Hand	4.50	.051	0.23	1.91	.187	0.11			
Task*Hand	0.88	.364	0.06	0.30	.589	0.02	0.09	.772	0.01
Grip*Hand	0.13	.725	0.01	1.00	.334	0.06	0.04	.852	0.00
Mode*Task*Grip	0.03	.863	0.00	9.77 **	.007	0.39			
Mode*Task*Hand	0.00	.984	0.00	1.26	.280	0.08			
Mode*Grip*Hand	0.05	.823	0.00	1.86	.193	0.11			
Task*Grip*Hand	4.61 *	.049	0.23	0.73	.407	0.05	0.25	.628	0.02
Mode*Task*Grip*Hand	6.15 *	.025	0.29	1.13	.305	0.07			

Note. The table gives Experiment A effect size and significance for main effects and their interactions. All factors are within-subjects factors. F-values with ps $< .05$ are marked *, with ps $< .01$ are marked **, with ps $< .001$ are marked ***. Effect sizes are reported as partial eta squared (η_p^2) values. RT = reaction time, MT = movement time, rotTime = rotation time.

Table S4. Experiment B Full Factorial ANOVA Results

Effect	RT			MT			rotTime		
	$F(1, 23)$	p	η_p^2	$F(1, 23)$	p	η_p^2	$F(1, 22)$	p	η_p^2
Intercept	549.71 ***	.000	0.96	910.48 ***	.000	0.98	347.86 ***	.000	0.94
Condition	15.52 ***	.001	0.40	2.00	.170	0.08	0.27	.609	0.01
Task	20.77 ***	.000	0.47	2.47	.130	0.10	0.03	.858	0.00
Grip	8.26 **	.009	0.26	71.42 ***	.000	0.76	99.42 ***	.000	0.82
Hand	1.15	.295	0.05	8.39 **	.008	0.27	2.98	.099	0.12
Condition*Task	0.24	.632	0.01	7.55 *	.011	0.25	1.71	.204	0.07
Condition*Grip	0.76	.393	0.03	1.05	.315	0.04	4.04	.057	0.16
Task*Grip	1.14	.296	0.05	0.15	.704	0.01	0.28	.603	0.01
Condition*Hand	0.02	.878	0.00	2.88	.103	0.11	3.24	.086	0.13
Task*Hand	1.71	.203	0.07	0.29	.597	0.01	0.54	.470	0.02
Grip*Hand	0.42	.521	0.02	14.02 **	.001	0.38	13.83 **	.001	0.39
Condition*Task*Grip	0.07	.788	0.00	2.51	.127	0.10	0.17	.685	0.01
Condition*Task*Hand	0.38	.543	0.02	0.02	.889	0.00	0.25	.621	0.01
Condition*Grip*Hand	0.02	.898	0.00	0.83	.371	0.03	2.61	.120	0.11
Task*Grip*Hand	2.83	.106	0.11	0.18	.674	0.01	0.32	.577	0.01
Condition*Task*Grip*Hand	0.00	.963	0.00	1.35	.256	0.06	0.08	.784	0.00

Note. The table gives Experiment B effect size and significance for main effects and their interactions. All factors are within-subjects factors. F-values with ps $< .05$ are marked *, with ps $< .01$ are marked **, with ps $< .001$ are marked ***. Effect sizes are reported as partial eta squared (η_p^2) values. RT = reaction time, MT = movement time, rotTime = rotation time.

REFERENCES

Andrieu, P., Bonnans, V., Meneses, J., Millot, J.-L., Moulin, T., and Gharbi, T. (2014). A modular, computer-controlled system for olfactory stimulation in the mri environment. *Behavior research methods* 46, 178–184

D'Ausilio, A. (2012). Arduino: A low-cost multipurpose lab equipment. *Behavior research methods* 44, 305–313

Escobar, R. and Pérez-Herrera, C. A. (2015). Low-cost usb interface for operant research using arduino and visual basic. *Journal of the experimental analysis of behavior* 103, 427–435

Hollinger, A., Steele, C., Penhune, V., Zatorre, R., and Wanderley, M. (2007). fmri-compatible electronic controllers. In *Proceedings of the 7th international conference on New interfaces for musical expression* (ACM), 246–249

Pineño, O. (2014). Arduipod box: A low-cost and open-source skinner box using an ipod touch and an arduino microcontroller. *Behavior research methods* 46, 196–205

Plant, R. R. and Turner, G. (2009). Millisecond precision psychological research in a world of commodity computers: New hardware, new problems? *Behavior Research Methods* 41, 598–614

Rorden, C. and Hanayik, T. (2014). StimSync: Open-source hardware for behavioral and MRI experiments. *Journal of neuroscience methods* 227, 90–99

Schubert, T. W., D'Ausilio, A., and Canto, R. (2013). Using arduino microcontroller boards to measure response latencies. *Behavior research methods* 45, 1332–1346

Ulrich, R. and Giray, M. (1989). Time resolution of clocks: Effects on reaction time measurement-good news for bad clocks. *British Journal of Mathematical and Statistical Psychology* 42, 1–12
